# A Bifunctional Spin Label for Ligand Recognition on Surfaces

**DOI:** 10.1002/anie.201703929

**Published:** 2017-07-06

**Authors:** Michael A. Hollas, Simon J. Webb, Sabine L. Flitsch, Alistair J. Fielding

**Affiliations:** ^1^ Department of Chemistry Photon Science Institute University of Manchester Oxford Road Manchester M13 9PL UK; ^2^ Department of Chemistry Manchester Institute of Biotechnology University of Manchester 131 Princess Street Manchester M1 7DN UK

**Keywords:** carbohydrates, EPR spectroscopy, nanoparticles, sensors, spin labels

## Abstract

In situ monitoring of biomolecular recognition, especially at surfaces, still presents a significant technical challenge. Electron paramagnetic resonance (EPR) of biomolecules spin‐labeled with nitroxides can offer uniquely sensitive and selective insights into these processes, but new spin‐labeling strategies are needed. The synthesis and study of a bromoacrylaldehyde spin label (BASL), which features two attachment points with orthogonal reactivity is reported. The first examples of mannose and biotin ligands coupled to aqueous carboxy‐functionalized gold nanoparticles through a spin label are presented. EPR spectra were obtained for the spin‐labeled ligands both free in solution and attached to nanoparticles. The labels were recognized by the mannose‐binding lectin, Con A, and the biotin‐binding protein avidin‐peroxidase. Binding gave quantifiable changes in the EPR spectra from which binding profiles could be obtained that reflect the strength of binding in each case.

The development of selective and non‐perturbing molecular probes is vital for understanding the complex interactions of biological systems with biomolecules on surfaces. Nitroxide radical spin labels for electron paramagnetic resonance (EPR) spectroscopy are an effective way to monitor protein dynamics.^1^, ^2^, ^3^ By using nitroxide spin labels, binding of various enzymes,^4^ lectins,^5^ RNA,^6^ and other bio‐macromolecules may be observed and quantified with EPR, offering opportunities to understand interfacial recognition at surfaces, including how receptor density on cell surfaces affects multivalent interactions with proteins.

Studies on bioactive ligands immobilized on nanomaterials have shown that substrate density has a dramatic effect on the enzymatic reaction kinetics on a surface.^7^ Similarly some proteins show an improvement in binding with greater ligand density^8^ while others show a decrease.^9^ For example, the tetravalent mannose binding lectin concanavalin A (Con A), has an affinity for clustered membrane bound mannose moiety lipids 3‐fold weaker than it has in solution.^9^

Measuring multivalent binding on surfaces can be challenging. Controlling and/or quantifying ligand density on a surface is often overlooked, assumed, or too difficult to measure. EPR can make valuable contributions to this area. One approach would be to create small bifunctional spin labels that can be placed between a biologically active ligand and a surface (Figure 1). This approach avoids significant changes of the ligand binding characteristics whilst tailoring the conformational freedom provided to the spin label. By monitoring changes in the EPR spectrum during protein binding, such a system may allow investigation of enzymatic reactions at surfaces, inform on the degree of clustering on surfaces, and give insight into the effect of ligand density on protein binding. Currently, there are no examples of spin labels applied to link a wide variety of bioactive ligands to surfaces (Figure 1).

**Figure 1:**
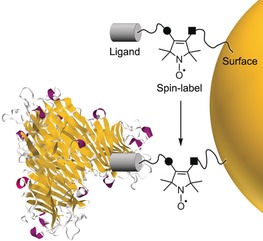
The concept of using a bifunctional spin label for studying surface‐ligand binding to proteins.

The bromoacrylaldehyde spin label (BASL) **3** fits the above requirements: it is small, with the structure stripped down to the essential functional groups required: a stable nitroxide spin label, an electrophilic carbon to allow ligation to nucleophiles and an aldehyde for reductive aminations. These functional groups are highly compatible with established coupling techniques, both to linkers and surfaces.^10^, ^11^, ^12^, ^13^, ^14^ We found that pyrrolidine aldehyde **3** (Figure 2 a) had been reported previously; however, its dual functionality was not exploited in spin labeling.^15^ Our first aim was to establish a synthetic route that would yield reliable gram quantities of fully characterized material for subsequent studies.

**Figure 2:**
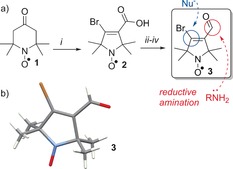
a) Synthesis of bifunctional spin label **3**. i) NaOH, Br_2_, H_2_O then Na_2_S_2_O_3_; ii) SOCl_2_, diethyl ether, −78 °C; iii) NaHCO_3_, NaBH_4_, 1,4‐dioxane; iv) pyridinium dichromate, CH_2_Cl_2_. b) X‐ray crystal structure of **3**.

Tri‐bromination and Favorskii rearrangement of commercially available 4‐oxo‐TEMPO **1** (Figure 2 a),^16^ afforded the pyrrolidine nitroxide **2**. Then, acid chloride formation and subsequent reduction with sodium borohydride gave an alcohol^17^ that was selectively oxidized with pyridinium dichromate to give BASL **3** in gram quantities. Owing to the paramagnetism of nitroxide radicals, NMR analysis is not possible and EPR gives little information on the molecular structure of the spin label. Therefore, to unambiguously characterize aldehyde **3**, the crystal structure was obtained (Figure 2 b).

With gram quantities of **3** in hand, this compound was tested as a bifunctional label. Sugars bearing amino‐,^18^ azido‐ (for click chemistry),^19^ and thiol^20^ reactive handles are commonly used for biofunctionalization. Given that the addition of thiols to **3** had already been demonstrated,^15^ BASL **3** was reacted with thiol terminated 2‐thioethyl‐α‐d‐mannose and 2‐thioethyl‐d‐biotin amide to give the spin‐labeled ligands **4** and **5**, respectively (Scheme 1), which could be purified by preparative high‐performance liquid chromatography (HPLC).

**Scheme 1:**
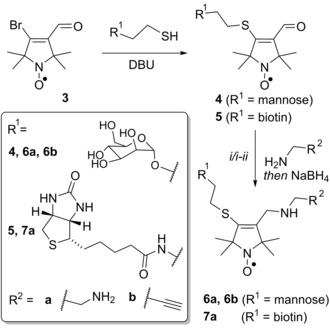
Sequential addition of functional groups to bifunctional spin label **3**. DBU=1,8‐diazabicyclo[5.4.0]undec‐7‐ene. If R^1^=mannose: i) H_2_NCH_2_R^2^, then NaBH_4_; if R^1^=biotin: i) H_2_N(CH_2_)_2_NHFmoc, then NaBH_4_; ii) piperidine.

These spin‐labeled ligands can be ligated to primary amines by reductive amination. This reactivity was exemplified with three reagents: ethylenediamine and mono‐FMOC ethylenediamine, to give amino‐functionalized products that can be ligated to carboxylic acid terminated surfaces, and propargylamine, which installs an alkyne group for click‐chemistry applications. Coupling in each case was achieved by reductive amination using sodium borohydride in methanol, yielding the amine terminated radicals **6 a** and **7 a**, and the alkyne‐terminated sugar radical **6 b** (Scheme 1).

To assess whether spin labels **6** and **7** could report on binding events with multivalent proteins, a mannose‐recognizing lectin (concanavalin A) and two biotin‐recognizing proteins (streptavidin, avidin‐peroxidase) were employed, selected to exemplify either weak or strong binding, respectively. Continuous‐wave (CW) EPR spectra were obtained for the spin‐labeled mannose aldehyde **4** (100 μm) in 0.01 m potassium borate buffer (Figure 3 a inset), before and after titration with concanavalin A (Con A, 25–200 μm). When the spectra from the titration were each normalized by setting the mid‐field line height to one, a decrease in the high‐field line height was observed that could be plotted against Con A binding site concentration. Fitting the data to a 1:1 binding model (sugar:lectin binding site) using DynaFit^21^ (Figure 3 a) gave an apparent dissociation constant (*K*_d_) for the mannosyl spin label **4** to each subunit of Con A of about 0.1 mm, close to the value for methyl mannoside (*K*_d_=0.13 mm).^22^ The value of this binding constant indicates that the mannose spin label **4** is recognized by Con A without significant perturbation of binding affinity. Subsequent addition of mannose to the mixture with the highest Con A concentration (200 μm) reversed both the broadening and hyperfine changes (Supporting Information, Figure S7). This was ascribed to displacement of the spin‐labeled mannose by free mannose and indicated that the changes in lineshape observed were due to Con A binding rather than changes in viscosity or polarity.

**Figure 3:**
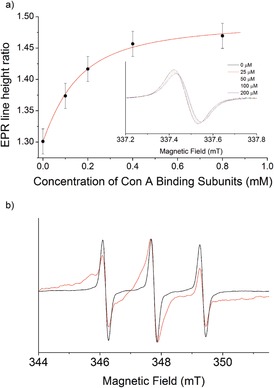
a) 1:1 Binding curve with apparent *K*_d_ of 0.1 mm, fitted to the change in the central/high‐field line height ratio on addition of Con A to **4**. Inset: Normalized CW EPR spectra of **5** (100 μm) with varying amounts of Con A (high field only). Estimated error is ±0.02. b) Normalized CW EPR spectra of the free biotin spin label **5** (2 μm) with (red) and without (black) streptavidin (1.8 μm).

CW EPR spectra were also obtained for the spin‐labeled biotin aldehyde **5** (2 μm) with and without streptavidin (1.8 μm) in water (Figure 3 b). Simulations (Supporting Information, Figure S12) indicated that the population of bound species was about 95 %. In contrast to the Con A binding, there was significant broadening of all three resonances upon addition of streptavidin, most likely due to tight binding to streptavidin leading to a significant reduction in molecular motion, whereas the binding site of Con A is known to be particularly flexible.^23^

Gold nanoparticles can be utilized as surfaces for the study of enzymatic reactions,^24^ where their large surface area is a particular advantage. The ability of gold nanoparticles to present highly tailored surfaces, using self‐assembled monolayers (SAMs) of alkyl thiolates to display desired functionality, allows nanoparticles to mimic biomacromolecules and creates useful artificial platforms in water for the study biological systems.^25^ While the EPR spectra of nitroxides on gold nanoparticles is known,^26^ this work has been conducted exclusively in non‐polar solvents, and therefore unsuitable for in vivo experiments.

The amino‐functionalized conjugates **6 a** and **7 a** were directly ligated onto carboxy‐terminated SAMS on gold surfaces. Gold nanoparticles were created using a modified Frens procedure^27^ to give citrate capped nanoparticles about 20 nm in size. Citrate capped nanoparticles were passivated for 24 hours with excess alkyl thiol (1:1 carboxylic acid/polyethylene glycol terminated thiols). To remove any residual thiol, the nanoparticles were then dialyzed. Peptide coupling of the amine terminated spin labels **6 a** and **7 a** to functionalized Au nanoparticles was achieved using 1‐ethyl‐3‐(3‐dimethylaminopropyl) carbodiimide (EDC) and *N*‐hydroxysulfosuccinimide (s‐NHS) (Scheme 2).^28^ After stirring for 24 hours the nanoparticles were sonicated and re‐dialyzed to remove any unbound spin label, giving purified spin‐labeled Au nanoparticles.

**Scheme 2:**
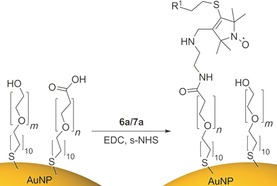
Synthesis of gold nanoparticles spin‐labeled with **6 a**/**7 a**. *m*=3 and *n*=6.

CW EPR spectra were obtained for the spin‐labeled Au nanoparticles (Figure 4 inset; Supporting Information, Figures S8–S10). When compared to the free spin labels in water, there was observable broadening for both biotin and mannose functionalized nanoparticles, indicating reduced molecular motion of the spin labels. A greater change in the lineshape might have been expected when attaching a spin label to a gold nanoparticle; however, the long PEG linkers of the carboxy‐terminated alkyl thiols appear to permit significant motion. The dynamics of labeled alkyl thiols on gold nanoparticles has previously been investigated, showing that the length of the spin‐labeled thiol relative to the unfunctionalized thiol has the largest effect on the dynamics.^29^ It was hypothesized that this design feature would be beneficial for investigating the binding of multivalent proteins, as subsequent complexation to proteins may produce a greater change of the lineshape.

**Figure 4:**
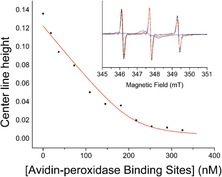
1:1 Binding curve with *K*_d_ of 8 nm fitted to the change in line height of the mid‐field resonance of the biotin spin‐labeled nanoparticles ([spin label]=210 nm) against avidin binding site concentration (calculated by UV/Vis spectrometry). Inset: CW EPR spectra of biotin functionalized nanoparticles (red) compared with the free biotin spin label **7 a** (black), and nanoparticles treated with 400 nm avidin‐peroxidase (blue).

Addition of Con A (up to 100 μm) to the mannose‐functionalized nanoparticles led to a color change and observable agglomeration of the nanoparticles over time, showing that the surface‐immobilized mannosyl spin label remains available to the lectin. This agglomeration was reversible, with addition of excess mannose allowing the nanoparticles to be re‐suspended and resulting in the recovery of the original surface plasmon resonance band (Supporting Information, Figure S4). EPR spectra obtained demonstrated recognition by Con A; however, a binding profile could not be produced, owing to the precipitation of the particles. The EPR spectral line shape was not influenced by this agglomeration process, as the spin labels only report on the local dynamics of the individual spin labels. To avoid similar agglomeration upon addition of streptavidin to biotin‐functionalized nanoparticles, avidin‐peroxidase was used. It was hoped that the presence of the conjugated peroxidase would hinder the agglomeration process.

In contrast to the addition of Con A to mannose functionalized nanoparticles, addition of avidin‐peroxidase (0–100 nm) to the biotin functionalized nanoparticles led to no observable color change or agglomeration. Previous studies suggested that agglomeration of biotinylated nanoparticles by avidin‐peroxidase is slow and unfavorable, leading only to small clusters and no precipitation.^30^ The CW EPR spectra showed significant broadening on addition of avidin‐peroxidase (Figure 4 inset). This broadening led to the decreasing intensity of all three nitroxide resonances, which could be monitored quantitatively (Figure 4). Fitting of two sets of data to a 1:1 complexation model using DynaFit^21^ (per avidin binding site) gave an average *K*_d_ value of approximately 9.2 nm with a standard deviation of 1.55 nm. This is much higher than biotin complexation by avidin in solution (*K*_d_≈10^−6^ nm), but consistent with reports of weakened binding caused by steric hindrance at the surface of the nanoparticle.^31^

In conclusion, a new synthetic strategy has been demonstrated that uses spin labels to link biofunctional groups to surfaces. BASL **3** has been synthesized in gram quantities and shown to undergo Michael addition/elimination with thiol‐terminated bioligands. Subsequent reductive amination of these products gave amino‐terminated biofunctional spin labels **6 a** and **7 a**, which could be attached to carboxy‐functionalized surfaces through peptide coupling. Similarly, alkyne‐terminated mannosyl spin label **6 b** was synthesized, which will allow spin labeling through click chemistry. CW EPR spectroscopy was able to report on the recognition of mannosyl spin label **4** by the lectin Con A and the biotin spin label **5** by streptavidin. Aqueous gold nanoparticles functionalized with either label **6 a** or label **7 a** were also recognized by Con A and avidin‐peroxidase, respectively. Reversible nanoparticle agglomeration by Con A was observed for the mannose functionalized nanoparticles. However, little agglomeration was observed upon mixing biotin‐functionalized nanoparticles with avidin‐peroxidase, and EPR provided a binding profile. To our knowledge, this is the first applied example of a spin label that can link a wide variety of bioactive ligands to surfaces, providing a new platform for future investigations of multivalent protein interactions with surface‐bound ligands and substrates. Furthermore, with recent advances in in vivo EPR imaging technology, spin‐labeled bioactive ligands linked to therapeutic nanostructures could be an exciting new class of theranostic agents.^32^

## Conflict of interest

The authors declare no conflict of interest.

## Supporting information

As a service to our authors and readers, this journal provides supporting information supplied by the authors. Such materials are peer reviewed and may be re‐organized for online delivery, but are not copy‐edited or typeset. Technical support issues arising from supporting information (other than missing files) should be addressed to the authors.

Supplementary
