# Supplementary material for: A Bifunctional Spin Label for Ligand Recognition on Surfaces
Source: Angew Chem Int Ed Engl. 2017 Jul 6;56(32):9449–53. doi: 10.1002/anie.201703929 (PMC5577508; doi:10.1002/anie.201703929)
Supplement: Supplementary file 1 — Supplementary [file ANIE-56-9449-s001.pdf]

Angewandte Chemie
Eine Zeitschrift der Gesellschaft Deutscher Chemiker

Supporting Information

A Bifunctional Spin Label for Ligand Recognition on Surfaces

Michael A. Hollas, Simon J. Webb, Sabine L. Flitsch, and Alistair J. Fielding**

anie_201703929_sm_miscellaneous_information.pdf

Supporting Information

Table of Contents

1. Synthesis 2

1.1 Instrumentation, materials and general methods 2

1.2 Nitroxide synthesis 2

1.3 2-thioethyl-α-D-mannoside synthesis 5

1.4 2-Thioethyl-N-D-biotin synthesis 8

1.5 Spin labelled ligand synthesis 10

1.6 Gold nanoparticles synthesis 14

2. EPR and NMR 18

2.1 General EPR 18

2.2 Mannose/Con A EPR 18

2.3 Biotin/Streptavidin EPR 26

2.4 NMR of radicals 34

3. Simulation Code 34

4. References 36

1. Synthesis

1.1 Instrumentation, materials and general methods

Nuclear Magnetic Resonance (NMR) spectra were recorded on a Bruker Ultrashield 400 MHz spectrometer. 1H and 13C spectra were referenced relative to the solvent residual peaks and chemical shifts (δ) reported in ppm downfield of tetramethylsilane ($CDCl_3$ δ H: 7.26 ppm, δ C: 77.16 ppm; CD_3OD δ H: 3.31 ppm, δ C 49.00 ppm). Splitting patterns are abbreviated as follows: singlet (s), doublet (d), triplet (t), multiplet (m), broad (br) or some combination of these. X-ray crystallography was recorded using a Supernova four-circle diffractometer. Flash column chromatography was carried out using Aldrich silica gel 60 Å, 230-400 Mesh. Thin layer chromatography (TLC) was performed using commercially available precoated plates (Macherey-Nagel, POLYGRAM®. SIL G/UV254) and visualized with UV light at 254 nm. Reverse phase HPLC was carried out using a 250 x 21.2 mm Phenomedia C18 5-micron column at a flow rate of 15 mL/min. All solvents were obtained from Sigma Aldrich and used as received. Per-deuterated solvents for NMR spectroscopy were obtained from Sigma Aldrich. Alkyl thiolates were obtained from Prochimia Surfaces (Poland). All other commercially available chemicals were obtained from either Aldrich or Fisher, unless indicated otherwise. Water used for nanoparticle synthesis was HPLC grade obtained from Sigma Aldrich.

1.2 Nitroxide synthesis

1.2.1 3-Bromo-4-carboxy-2,2,5,5-tetramethyl-1H-pyrrol-1-yloxyl radical

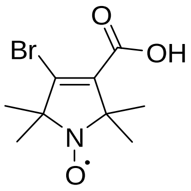

2

Bromine (2.1 mL, 81 mmol) was added dropwise, under a nitrogen atmosphere, to a stirring solution of water (60 mL) and NaOH (7.84 g, 49 mmol) at -5 °C, after which; a solution of 4-oxo-TEMPO **1** (2.0 g, 11.8 mmol) and NaOH (1.97 g, 12.3 mmol) in a mixture of water (39.2 mL) and 1,4-dioxane (9.8 mL),

cooled to -10 °C, was added slowly over 10 min The reaction was left stirring for 20 min at -2 °C, after which a solution of $Na_2S_2O_3{\cdot}5H_2O$ (0.8 g) in water (2.4 mL) was added. The reaction was heated to 30 °C, stirred for 30 min, cooled to -2 °C, filtered, and the precipitate washed with water. The filtrate was acidified with conc. HCl to pH 2-3 at -2 °C, filtered and washed with cold water; yielding 3-bromo-4-carboxy-2,2,5,5-tetramethyl-1H-pyrrol-1-yloxyl radical (1.03 g, 33 %) as a pale yellow solid. Recrystallization with boiling IPA gave the 3-bromo-4-carboxy-2,2,5,5-tetramethyl-2,5-dihydropyrroloxyl **2** (0.411 g, 13 %) as a yellow solid: mp. 230-231 °C [lit 232-233.5 °C][1]; Anal. Calcd for $C_9H_{13}BrNO_3$: C, 41.08; H, 4.98; N, 5.32. Found: C, 40.72; H, 4.87; N, 5.21; *v* (cm^{-1}) 1610 (C=C), 1716 (C=O); *m/z* (ESI+) 261, 263 (M-H).

1.2.2 3-Bromo-4-carboxychloride-2,2,5,5-tetramethyl-1H-pyrrol-1-yloxyl radical

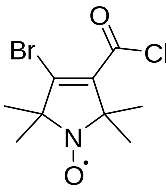

8

Pyridine (0.42 mL, 5.1 mmol), then thionyl chloride (0.87 mL, 12.3 mmol) in dry diethyl ether (12 mL) were added to a suspension of 3-bromo-4-carboxy-2,2,5,5-tetramethyl-2,5-dihydropyrroloxyl **2** (0.54 g, 2.1 mmol) in dry diethyl (12 mL) at -78 °C under N_2, over 15 min. The reaction was allowed to warm to 0 °C and stirred for 2 h at 0 °C and then 2 h at room temperature. The residue was filtered, washed with toluene, and the filtrate evaporated under vacuum; yielding the acid chloride **8** (0.48 g, 83 %) as orange/yellow crystals: *v* (cm^{-1}) 2980, 2931 (Me), 1719 (C=O), 1610 (C=C), 1364 (N-O), 571 (C-Br).

1.2.3 3-Bromo-4-hydroxymethyl-2,2,5,5-tetramethyl-1H-pyrrol-1-yloxyl radical

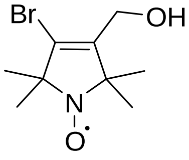

9

$NaHCO_3$ (0.68 g, 8.1 mmol) and $NaBH_4$ (0.77 g, 20.2 mmol) were added in portions over 5 min to a solution of the acid chloride **8** (1.14 g, 4.0 mmol) in dioxane (40 mL) at 0 °C. The reaction was then stirred for 3 h at room temperature, after which 20 mL sat. Na_2CO_3 was added, the reaction poured into 60 mL water and extracted with chloroform (3 x 30 mL). The organic phases were combined, washed with brine, dried over $MgSO_4$, and evaporated under vacuum to give **9** (0.81 g, 80 %) as yellow crystals; mp. 151 °C [lit 152-153 °C][2]; *v* (cm^{-1}) 3373 (br, OH), 2980, 2935 (Me), 1648 (C=C), 1424 (C-O), 1362 (N-O), 565 (C-Br); m/z (ESI+) 249.0 ($M+H^+$, 100 %).

1.2.4 3-Bromo-4-formyl-2,2,5,5-tetramethyl-1H-pyrrol-1-yloxyl radical

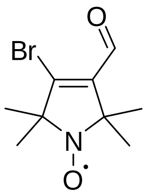

3

PDC (1.81 g, 4.8 mmol) was added to a stirring solution of the alcohol radical **9** (0.80 g, 3.2 mmol) in DCM (40 mL) under N_2. The suspension was stirred for 48 h at room temperature, after which no starting material was observed by TLC. Diethyl ether (100 mL) was added and the mixture was poured into 50 mL of water. The organic phase was separated, washed with $NaHCO_3$ (3 x 50 mL), and collected. The aqueous phase was extracted with ether (3 x 20 mL) and the organic phases combined and washed with brine (50 mL). The combined organic phases were filtered through Celite©, dried ($MgSO_4$), and evaporated under vacuum. Purification by flash chromatography (10 % EtOAc in cyclohexane) yielded the 3-bromo-aldehyde **3** (0.48 g, 60 %) as bright yellow crystals: mp. 125 °C [lit 123-124 °C][2]; *v* (cm^{-1}) 2979, 2933 (Me), 1679 (C=O), 1604 (C=C), 1364 (N-O), 556 (C-Br). CCDC 1431285 contains the supplementary crystallographic data for this paper. This data is provided free of charge by The Cambridge Crystallographic Data Centre.

1.3 2-thioethyl-α-D-mannoside synthesis

1.3.1 1,2,3,4,6-Penta-*O*-aceto-α/β-D-mannose

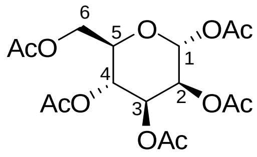

10

D-mannose (5.0 g, 28 mmol) was added slowly to a stirring solution of Ac_2O (15 mL, 159 mmol) with 2 drops of H_2SO_4 at -5 to 0 °C. The addition of mannose was controlled to avoid the reaction temperature rising above 40 °C. The reaction was stirred for 30 min, concentrated under vacuum, the residue dissolved in EtOAc (50 mL), and water (100 mL) added. The aqueous phase was extracted with EtOAc, the organic phases combined, washed with sat. $NaHCO_3$ solution (100 mL), brine (100 mL), dried ($MgSO_4$), and evaporated under vacuum. The residue was dissolved in diethyl-ether and quickly dried under vacuum, producing a white foam **10** (8.9 g, 81.7 %): δ_H (400 MHz, $CDCl_3$, mixture of α and β anomers), α anomer: 2.01, 2.05, 2.09, 2.17, 2.18 (s, 5 x 3H, $COCH_3$), 4.01-4.07 (m, 1H, H5), 4.10 (dd, 1H, H6-a, *J* = 12.4, 2.5 Hz), 4.29 (dd, 1H, H6-b, *J* = 12.4, 4.8 Hz), 5.13 (dd, 1H, H3, *J* = 9.9, 3.3 Hz), 5.34-5.36 (m, 2H, H3, H4), 6.09 (d, 1H, H1, *J* = 2.0); β anomer: 2.01, 2.03, 2.07, 2.15, 2.16 (s, 5 x 3H, $COCH_3$), 3.79 (ddd, 1H, H5, *J* = 9.9, 5.3, 2.4 Hz), 4.11 (dd, 1H, H6-a, *J* = 12.4, 2.5 Hz), 4.28 (dd, 1H, H6-b, *J* = 12.4, 5.3 Hz), 5.11 (dd, 1H, H3, *J* = 9.9, 3.3 Hz), 5.27 (t, 1H, H4, *J* = 10 Hz), 5.46 (dd, 1H, H2, 3.3, 1.2 Hz), 5.84 (d, 1H, H1, *J* = 1.2 Hz); m/z (ESI+) 391 (M+H, 100 %).

1.3.2 2-Bromoethyl-2,3,4,6-tetra-*O*-acetyl-α-D-mannopyranoside

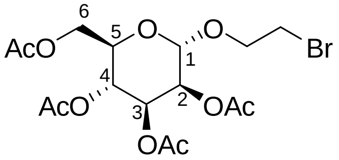

11

BF_3Et_2O (4.9 mL, 38.6 mmol) was added dropwise over 15 min to a solution of 1,2,3,4,6-penta-O-acetyl-α-D-mannose **10** (3 g, 7.7 mmol) and $Br(CH_2)_2OH$ (0.67 mL, 9.4 mmol) in DCM (15 mL) at 0 °C under N_2. After 1 h the solution was warmed to room temperature. After 25 h the reaction solution was

added to ice water (20 mL) and extracted with DCM (20 mL x 2). These extracts were combined, washed with water (20 mL), sat. $NaHCO_3$ (aq. 20 mL), water (20 mL), dried ($MgSO_4$), filtered and the solvent removed under vacuum. The residue was crystallized from EtOAc/hexane to give 2-bromoethyl 2,3,4,6-tetra-*O*-acetyl-D-mannopyranoside **11** (1.68 g, 48 %): mp. 118-119 °C [lit 117-119 °C][3]; v (cm^{-1}) 1753 (C=O), 1368 (C-O ester), 1223 (C-O ether), 1042 (C-O), 599 (C-Br); δ_H (400 MHz, $CDCl_3$) 2.00, 2.06, 2.11, 2.16 (s, 4 x 3H, $COCH_3$), 3.52 (t, 2H, CH_2Br, J = 5.8 Hz), 3.86-3.92 (m, 2H, CH_2O), 4.95-4.01 (m, 1H, H5), 4.11-4.17 (m, 2H, H6), 4.28 (dd, 1H, H2, J = 12.6, 5.8 Hz), 4.88 (d, 1H, H1, J = 1.8 Hz), 5.26-5.30 (m, 1H, H3), 5.36 (dd, 1H, H4, J = 10.1, 3.5 Hz); δ_C (100 MHz, $CDCl_3$) 20.8, 20.9, 20.9, 21.0 (4 <u>Me</u>CO), 29.7 (CH_2Br), 62.5 (C6), 66.1 (C2), 68.6, 69.0, 69.2 (C-3,4,5), 76.8 (CH_2O), 97.9 (C1), 170.7, 170.2, 170.0, 169.9 (4 Me<u>C</u>O); *m/z* (ESI+) 477 (100 %, $M+Na^+$).

1.3.3 2-Thiocyanoethyl-2,3,4,6-tetra-O-acetyl-α-D-mannopyranoside

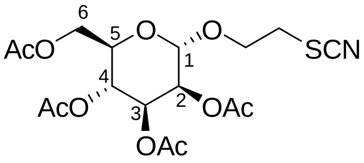

12

A solution of the halo-sugar **11** (1.00 g, 2.2 mmol), KSCN (0.98 g, 3.33 mmol) in DMF (4 mL), *n*-Bu_4NI (20 mg) and dibenzo-18-crown-6-ether (3.5 mg) was stirred at 80-90 ºC for 3-4 h. After cooling, Et_2O-toluene (1:3, 40 mL) was added and the mixture extracted with water (2 x 25 mL). The organic phase was dried ($MgSO_4$), evaporated under vacuum, and the residue purified by flash chromatography (EtOAc-hexane 1:2 to 1:1), yielding a colourless sticky oil **12** (0.87 g, 92 %); mp. 72-76 °C [lit 71-75 °C][3]; v (cm^{-1}) 1753 (C=O), 1368 (C-O ester), 1223 (C-O ether), 1042 (C-O), 599 (C-Br); δ_H (400 MHz, $CDCl_3$) 2.00, 2.06, 2.12, 2.17 (s, 4 x 3H, $COCH_3$), 3.17-3.23 (m, 2 H, CH_2S), 3.90 (dt, 1H, CH_2O, J = 10.9, 5.4 Hz), 4.00-4.10 (m, 2H, H5, CH_2O), 4.15 (dd, 1H, H6a, J = 12.2, 2.3 Hz), 4.31 (dd, 1H, H6, J = 12.2, 5.7 Hz), 4.90 (br s, 1H, H1), 5.24-5.38 (m, 3H, H2,3,4); δ_C (100 MHz, $CDCl_3$) 20.7, 20.8, 20.9, 21.0 (4 <u>Me</u>CO), 33.4 (CH_2S), 62.5 (C6), 65.9 (C4), 66.0 (CH_2O), 68.8, 69.3, 69.6 (C2,3,5), 97.8 (C1), 112.0 (SCN), 169.7, 169.9, 170.0, 170.7 (4 Me<u>C</u>O); m/z (ESI+) 456.1 (100 %, M+Na).

1.3.4 2-Thioethyl-2,3,4,6-tetra-*O*-acetal-α-D-mannopyranoside

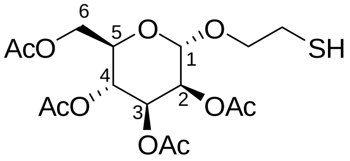

13

Zn dust (1.5 g) was added to a stirring solution of the thiocyanide acetylated sugar **12** (0.8 g, 1.85 mmol) in glacial AcOH (25 mL). The reaction mixture was kept under reflux for 3 h. After cooling, DCM (50 mL) was added and the solution filtered through Celite©. The filtrate was washed with water (25 mL) sat. $NaHCO_3$ solution (2 x 25 mL), the organic layer dried ($MgSO_4$) and evaporated under vacuum. The residue was purified by flash chromatography (EtOAc/cyclohexane 1:2), yielding the protected thio-sugar **13** (0.37 g, 49 %): mp. 86-88 °C [lit 86-89 °C][3]; ν (cm^{-1}) 1746 (C=O), 1370 (C-O ester), 1224 (C-O ether), 1047 (C-O); δ_H (400 MHz, $CDCl_3$) 1.56 (t, 1H, SH, *J* = 8.5 Hz), 2.00, 2.05, 2.11, 2.15 (s, 4 x 3H, CH_3CO), 2.74 (dt, 2H, CH_2S, *J* = 8.0, 6.5 Hz), 3.65 (dt, 1H, CH_2O, *J* = 10.1, 6.2 Hz), 3.81 (dt, 1 H, CH_2O, *J* = 10.1, 6.2 Hz), 4.08 (m, 1H, H5), 4.13 (dd, 1H, H6-a, *J* = 12.3, 2.4 Hz), 4.29 (dd, 1H, H6, *J* = 12.3, 5.4 Hz), 4.86 (d, 1H, H1, *J* = 1.4 Hz), 5.27 (dd, 1H, H2, *J* = 3.1, 1.4 Hz), 5.29 (t, 1H, H4, *J* = 9.7 Hz), 5.35 (dd, 1H, H3, *J* = 10, 3.1 Hz); δ_C (100 MHz, $CDCl_3$) 20.7, 20.8, 20.9, 20.9 (4 <u>Me</u>CO), 24.1 (CH_2S), 62.5 (C6), 66.2 (C4), 68.9, 69.1, 69.5 (C-2,3,5), 70.2 (CH_2O), 97.7 (C1), 169.8, 169.9, 170.1, 170.7 (4 Me<u>C</u>O); m/z (ESI+) 431.1 ($M+Na^+$, 100 %).

1.3.5 2-Thioethyl-α-D-mannopyranoside

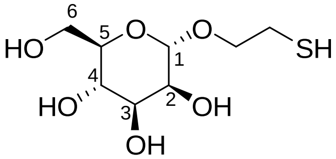

14

Sodium methanolate (2.5 mL, 0.2 M in MeOH) was added to the protected thio-sugar **13** (0.11 g) in dry methanol (11.5 mL), and the reaction stirred at room temperature for 1 h (verified by TLC). The mixture was initially dark red and on completion of the reaction was pale orange in colour. Dowex H+ ion exchange resin; first washed with methanol and dried over a Buchner funnel, was added to the basic

mixture until pH 7 as verified by Universal Indicator paper. The mixture was filtered and the solvent evaporated under vacuum, yielding the 2-thioethyl-α-D-mannose **14** as a cream coloured foam (0.16 g, quant); ν (cm^{-1}) 3369 (br, OH), 2478 (br, SH), 1450 (C-O), 1205 (C-O ether); δ_H (400 MHz, D_2O) 2.68 (m, 2H, CH_2S), 3.82-3.52 (m, 7H, CH_2O, H3-6), 3.88 (dd, 1H, H2, *J* = 3.5, 1.8 Hz), 4.82 (d, 1H, H1, *J* = 1.8 Hz); δ_C (100 MHz, D_2O) 23.3 (CH_2S), 60.9 (C6), 66.7 (C4), 69.0 (CH_2O), 69.9 (C2), 70.5; δ_c (100 MHz, D_2O) (C3), 72.8 (C5), 99.6 (C1); *m/z* (ESI+) 263.1 ($M+Na^+$, 100 %), 279.1 ($M+K^+$, 25 %).

1.4 2-Thioethyl-*N*-D-biotin synthesis

1.4.1 2,5-Dioxo-pyrrolidin-1-yl-D-biotin ester

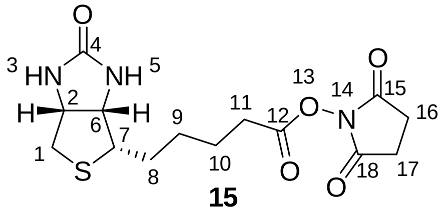

15

Biotin (0.31 g, 1.27 mmol) was dissolved in DMF (9 mL) with gentle warming (if required). On cooling to room temperature without precipitation, the solution was added to a stirring solution of DCC (0.26 g, 1.27 mmol) and pyridine (0.1 mL, 1.27 mmol). After 5 mins stirring, N-hydroxysuccinimide (NHS; 0.19 g, 1.65 mmol) was added and the reaction mixture was stirred at room temperature for 24 h. The product was filtered to remove any urea side products and concentrated under reduced pressure. The obtained solid was recrystallized using hot IPA, and washed with cold IPA to give the 2,5-dioxo-pyrrolidin-1-yl-D-biotin ester **15** as a white crystalline solid (0.304 g, 70 %); HRMS (ESI+) found 342.1132 ($M+H^+$), [$C_{14}H_{20}N_3O_5S+$] requires 342.1118; δ_H (400 MHz, D_6-DMSO) 6.41 (s, 1H, H-3), 6.35 (s, 1H, H-5), 4.31 (at, 1H, H-2), 4.15 (at, 1H, H-6), 3.12 (m, 1H, H-7), 2.86 (d, 1H, $H1_b$), 2.82 (s, 4H, H-16,17), 2.68 (t, 2H, H-11), 2.59 (d, 1H, $H1_a$), 1.74-1.37 (m, 6H, H-8-10); δ_c (100 MHz, D_6-DMSO) 170.2 (C4), 162.7 (C12), 168.9 (C15,18), 61.0 (C6), 59.2 (C2), 55.2 (C7), 30.0 (C11), 27.8,27.6 (C8-9), 25.4 (C16-17), 24.3 (C10).[4]

1.4.2 Bis((2-*N*-D-biotin) ethylamide)disulfide

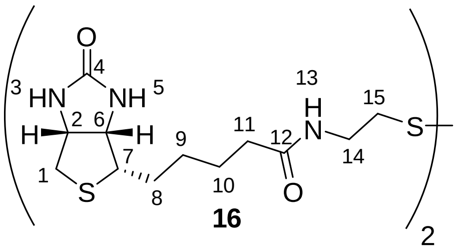

16

2,5-Dioxo-pyrrolidin-1-yl-D-biotin ester **15** (0.285 g, 0.83 mmol) was dissolved in DMF (10 mL) with gentle warming (if required). On cooling to room temperature without precipitation, an aqueous solution (1.5 mL) of cystamine dihydrochloride (0.10 mL, 0.46 mmol) and triethylamine (0.3 mL, 2.08 mmol) was added. The reaction mixture was stirred overnight (16 h) at room temperature after which the products were concentrated under reduced vacuum. The obtained solid was recrystallized using hot IPA, and washed with cold IPA to give the di-{(2-thioethyl)-biotin amide} **16** as a white crystalline solid (0.197 g, 78 %); HRMS (ESI+) found 605.1994 ($M+H^+$), [$C_{24}H_{41}N_6O_4S_4$+] requires 605.2067; δ_H (400 MHz, D_6-DMSO) 7.99 (t, 2H. H-13), 6.42 (s, 2H, H-3), 6.36 (s, 2H, H-5), 4.31 (at, 2H, H-2), 4.14 (at, 2H, H-6), 3.11 (m, 2H, H-7), 2.83 (dd, 2H, H-1_b), 2.77 (t, 4H, H-15), 2.58 (d, 2H, H-1_a), 2.08 (t, 4H, H-11), 1.68-1.24 (m, 12H, H-8-10); δ_c (100 MHz, D_6-DMSO) 172.2 (C4), 162.7 (C12), 61.0 (C6), 59.2 (C2), 55.4 (C7), 45.6 (C1), 37.9 (C14), 37.3 (C11), 35.1 (C15), 28.2, 28.0 (C8,9), 25.2 (C10).[5]

1.4.3 5-D-Biotin-(2-thioethyl) amide

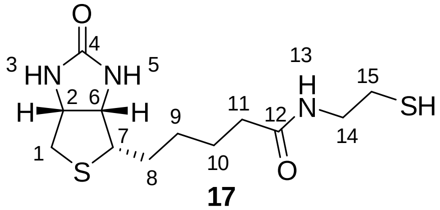

17

Di-{(2-thioethyl)-biotin amide} **16** (0.173 g, 0.29 mmol) was dissolved in DMF (10 mL) with gentle warming (if required). On cooling to room temperature without precipitation, DL-dithiothreitol (DTT; 0.133 g, 0.86 mmol) and TEA (3.6 µL, 0.02 mmol) were added and the reaction stirred at room temperature for 3 h. The products were concentrated under reduced vacuum and the remaining solid washed with DCM (10 mL x 3). Recrystallization with hot IPA yielded the 5-D-Biotin-(2-thioethyl) amide **17** as a white

crystalline solid (0.100 g, 57 %); HRMS (ESI+) found 304.1144 (M+H$^+$), [$C_{12}H_{22}N_3O_2S_2$+] requires 304.1148; δ_H (400 MHz, D_6-DMSO) 7.93 (t, 1H, H-13), 6.41 (s, 1H, H-3), 6.35 (s, 1H, H-5), 4.30 (at, 1H, H-2), 4.13 (at, 1H, H-6), 3.19 (adt, 2H, H-14), 3.10 (m, 1H, H-7), 2.83 (dd, 1H, H-1_b), 2.58 (d, 1H, H-1_a), 2.32 (t, 1H, SH), 2.07 (t, 2H, H-11), 1.68-1.24 (m, 6H, H-8-10); δ_c (100 MHz, D_6-DMSO) 172.0 (C14), 162.7 (C12), 61.0 (C6), 59.2 (C2), 55.4 (C7), 42.0 (C14), 35.1 (C11), 28.2 (C9), 28.0 (C15), 25.2 (C10), 23.5 (C8).[5]

1.5 Spin labelled ligand synthesis

1.5.1 3-(2-Thioethyl-α-D-mannose)-4-formyl-2,2,5,5-tetramethyl-1H-pyrrol-1-yloxyl Radical

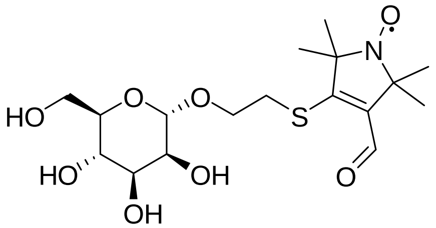

4

DBU (0.60 mL, 4.0 mmol) was added to a stirring solution of the 2-thioethyl-α-D-mannose **13** (0.294 g, 1.2 mmol) and the aldehyde radical **3** (0.302 g, 1.2 mmol) in DMF (5 mL) at room temperature. The reaction was stirred for 1 hour, after which no starting material was observed by TLC. The reaction was then purified by preparative reverse phase HPLC. The gradient profile was as follows: 5 % MeCN (in water) to 100 % MeCN over 40 min, with a retention time of 13.9 min for the sugar radical **4**, and 21.5 min for the radical aldehyde **3**. The solvents were removed using a vacuum centrifuge, yielding the sugar radical **4** as a yellow/orange oil (0.433 g, 87 %). HRMS (ESI+) found 429.1447 (M+H$^+$), [$C_{17}H_{28}NNaO_8S^{\cdot +}$] requires 429.1428; *v* (cm-1) 3313 (br, OH), 2976, 2930 (Me), 2861 (C-H), 1647 (C=O), 1607 (C=C), 1450 (C-O) 1355 (N-O).

1.5.2 3-(2-Thioethyl-α-D-mannose)-4-*N*-(2-aminoethyl)-2,2,5,5-tetramethyl-1H-pyrrol-1-yloxyl radical

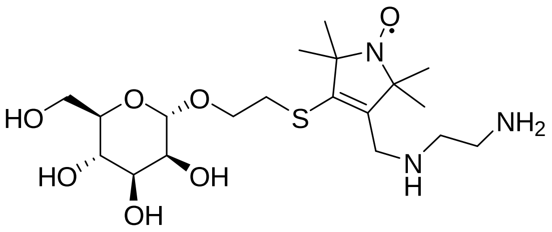

6a

Ethylene diamine (0.30 mL, 4.2 mmol) was added to a stirring solution of the spin labelled mannose **4** (0.0551 g, 0.14 mmol) in dry methanol (1 mL) at room temperature under N_2. The reaction was stirred for 30 min after which no starting material was observed by mass spectrometry, and a peak corresponding to the imine (*m/z* (ESI+) 449.2 ($M+H^+$)) appeared. $NaBH_4$ (8.2 mg, 0.22 mmol) dissolved in dry methanol (1 mL) was added to the solution, and the reaction followed by mass spectroscopy. After 1 hour the imine peak (*m/z* 449.2) had been replaced by the product **6a** (*m/z* (ESI+) 451.2 ($M+H^+$)). The reaction mixture was acidified with 10 % H_2SO_4 and purified by a cation exchange resin column (Dowex 50WX8 hydrogen form). The column was washed with water (2V) and 10 % H_2SO_4 (2V) before loading the crude mixture. It was then washed with water until the pH was neutral and the product was eluted off the column with 5 % NH_4OH. The solvents were removed using a vacuum centrifuge, yielding the amino functionalised spin labelled mannose **6a** as a yellow oil (0.0142 g, 23 %): HRMS (ESI+) found 451.2284 ($M+H^+$), [$C_{19}H_{37}N_3O_7S^{\cdot+}$] requires 451.2347; ν (cm-1) 3350 (br, OH), 2973, 2929 (Me), 2861 (C-H), 1630 (N-H), 1589 (C=C), 1475 (C-O), 1355 (N-O).

1.5.3 3-(2-Thioethyl-α-D-mannose)-4-*N*-(2-propargylamine)-2,2,5,5-tetramethyl-1H-pyrrol-1-yloxyl radical

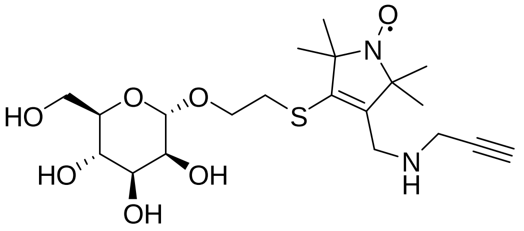

6b

Propargylamine (5 µL, 0.076 mmol) was added to a stirring solution of the spin labelled mannose **4** (24.3 mg, 0.06 mmol) in dry methanol (1 mL) at room temperature under N_2. The reaction was stirred for 30 min after which no starting material was observed by mass spectrometry and a peak corresponding to the imine (*m/z* (ESI+) 444.2 (M+H$^+$)). $NaBH_4$ (3.6 mg, 0.1 mmol) dissolved in dry methanol (1 mL) was added to the solution, and the reaction followed by mass spectroscopy. After 1 h the imine peak had been replaced by the product **6b** (*m/z* (ESI+) 446.2 (M+H$^+$)). The reaction mixture was acidified with 10 % H_2SO_4 and purified by a cation exchange resin column (Dowex 50WX8 hydrogen form). The column was washed with water (2V) and 10 % H_2SO_4 (2V) before loading the crude mixture. It was then washed with water until the pH was neutral and the product was eluted off the column with 5 % NH_4OH. The solvents were removed using a vacuum centrifuge, yielding the alkyne functionalised spin labelled mannose **6b** as a yellow oil (15.0 mg, 56 %): HRMS (ESI+) found 446.2067 (M+H$^+$), [$C_{20}H_{34}N_2O_7S^{\cdot+}$] requires 446.2081; v (cm^{-1}) 3367 br, OH), 3283 (C≡C), 2974, 2928 (C-H, Me), 2872 (C-H), 2123 (C-H, alkyne), 1611 (C=C), 1455 (C-O), 1372 (N-O), 1030 (s, br, C-O, alcohol).

1.5.4 3-(2-Thioethyl-D-biotin amide)-4-formyl-2,2,5,5-tetramethyl-1H-pyrrol-1-yloxyl Radical

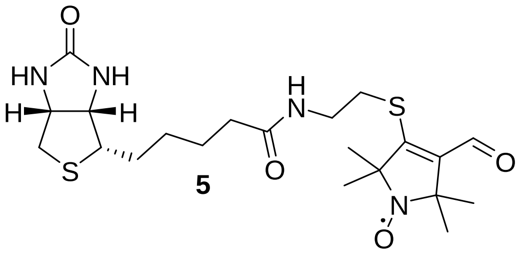

5

DBU (0.13 mL, 0.8 mmol) was added to a stirring solution of the 2-thioethyl-D-biotin amide **17** (0.080 g, 0.27 mmol) and the aldehyde radical **3** (0.0658 g, 0.27 mmol) in DMF (1 mL) at room temperature. The reaction was stirred for 1 h, after which no starting material was observed by TLC or MS. The reaction was then purified by preparative reverse phase HPLC. The gradient profile was as follows: 5 % MeCN (in water) to 100 % MeCN over 40 min, with a retention time of 15 min for the biotin radical **5**, and 19 min for the radical aldehyde **3**. The solvents were removed using a vacuum centrifuge, yielding the biotin radical aldehyde **5** as a yellow/orange oil (0.0156 g, 12 %): HRMS (ESI+) found 470.2021 (M+H$^+$), [$C_{21}H_{34}N_4O_4S_2^{\cdot+}$] requires 470.2016.

1.5.5 3-(2-Thioethyl-D-biotin amide)-4-*N*-(2-aminoethyl)-2,2,5,5-tetramethyl-1H-pyrrol-1-yloxyl radical

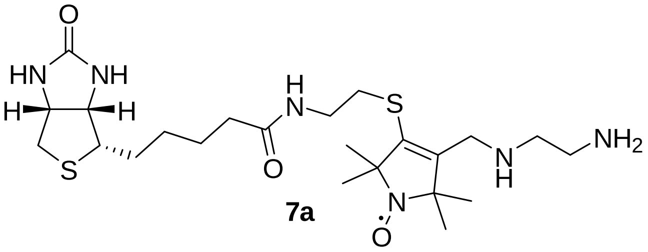

7a

Mono-Fmoc ethylene-diamine hydrobromide (12.0 mg, 0.033 mmol) was added to a stirring solution of biotin radical aldehyde **5** (15.6 mg, 0.033 mmol) in dry methanol (1 mL) at room temperature under N_2. The reaction was stirred for 1 h after which no starting material was observed by mass spectrometry. The reaction was then purified by preparative reverse phase HPLC. The gradient profile was as follows: 5 % MeCN (in water) to 100 % MeCN over 40 min, with a retention time of 22 min for the FMOC protected biotin radical imine**.** The solvents were removed using a vacuum centrifuge, and the residue dissolved in dry methanol (1 mL). $NaBH_4$ (2.0 mg, 0.053 mmol) was added to the stirring solution, and the reaction followed by mass spectroscopy. After 1 h the imine peak had been replaced by the amine (HRMS (ESI+) found 736.3447 (M+H$^+$), [$C_{38}H_{50}N_6O_5S_2^{\cdot+}$] requires 736.3279). The solvents were removed under vacuum; the residue dissolved in water (0.8 mL) and piperidine (0.2 mL) added. The reaction mixture was stirred for 1 h at room temperature, after which, the solid formed was removed by filtration and washed with water, and the filtrate concentrated under vacuum. The filtrate was acidified with 10 % H_2SO_4 and purified by a cation exchange resin column (Dowex 50WX8 hydrogen form). The column was washed with water

(2V) and 10 % H_2SO_4 (2V) before loading the crude mixture. It was then washed with water until the pH was neutral and the product was eluted off the column with 5 % NH_4OH. The solvents were removed using a vacuum centrifuge, yielding the biotin radical amine **7a** as a white solid (16.3 mg, 95 %): HRMS (ESI+) found 514.2769 ($M+H^+$), [$C_{23}H_{42}N_6O_3S_2^{\cdot+}$] requires 514.2754.

1.6 Gold nanoparticles synthesis

1.6.1 General synthesis

18 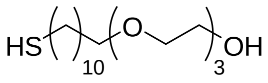

19 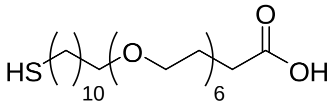

Gold nanoparticles were created using a modified Frens procedure[6] to give citrate capped nanoparticles ~20 nm in size (size may be roughly determined by position of the surface plasmon resonance band measured by UV – Figure S1).[7] $HAuCl_4 \cdot 3H_2O$ (0.0077 g) was dissolved in HPLC grade H_2O (1.54 ml, 0.01 % by weight). 1 ml of this gold solution (5 mg/mL) was added to 49 ml of HPLC grade H_2O and brought to reflux. 1 ml of trisodium citrate (10 mg/mL in HPLC grade H_2O) was added to the boiling gold solution and the mixture was allowed to reflux for 5 minutes at 140 ^oC under vigorous stirring. The solution was then cooled to room temperature.

Citrate capped nanoparticles were passivated with excess alkyl thiols (a mixture of polyethylene glycol **18** and carboxylic acid **19** terminated) and pH adjusted to > 11 with NH_4OH.[6] After 24 h stirring the nanoparticles were sonicated for ~5 min to break up any aggregated particles and dialyzed 3 x with 14K MWCO tubing to remove any residual akyl thiols. The nanoparticles were then dialyzed once more in 0.01 M potassium borate buffer, pH 9.

1.6.2 General procedure for peptide coupling on gold nanoparticles

To a stirring solution of thiol passivated nanoparticles (10 mL) a solution of the desired amine terminated spin label was added (200 µl, 1 mg/mL in 0.01 M potassium/sodium borate buffer). Solutions

of EDC (100 µl, 0.2 M) and s-NHS (200 µl, 0.2 M) were added simultaneously to the stirring nanoparticles. After stirring for 24 h the nanoparticles were sonicated and dialyzed 3 x with 14K MWCO tubing to remove any residual free spin label, giving purified spin labelled Au nanoparticles. These nanoparticles where then prepared for EPR studies by adding a known concentration of the respective binding protein and incubated for 10 mins.

1.6.3 Gold nanoparticle UV-Vis

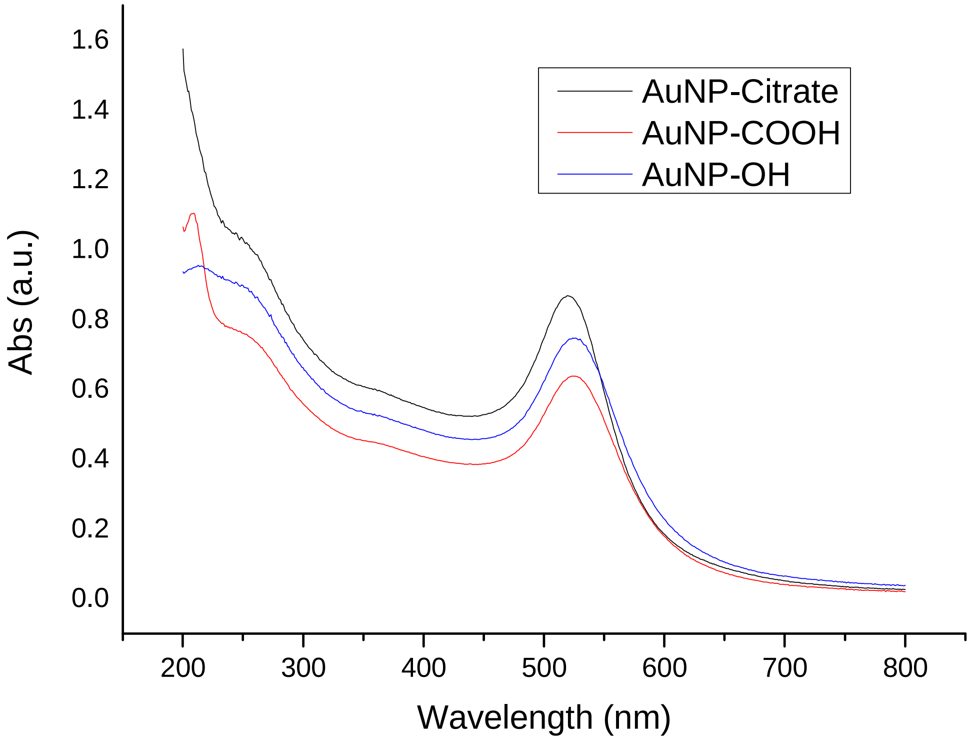

Figure S1: UV-Vis spectra of nanoparticles passivated with sodium citrate, PEG terminated alkyl thiols, and a mixture of PEG and carboxylic acid terminated alkyl thiols**.**

To investigate the avidity (agglomeration) of the mannose spin labelled nanoparticles with Con A, 1 mL samples of the nanoparticles were placed in Eppendorf micro centrifuge tubes, and varying amounts of a 0.1 mg/mL Con A solution was added, to give a range of concentrations (0-9.1 µg/mL). The nanoparticles were incubated at room temperature, and their UV-Vis spectra obtained at 3, 8, and 24 hours. Addition of Con A led to an observable colour change and agglomeration of the nanoparticles (Fig.

S2), but only for those treated with > 5 µg/mL Con A. The UV-Vis spectrum of the untreated nanoparticles showed a maximum absorbance at 530 nm, corresponding to the surface plasmon band.[8] On addition of Con A, there was a shift of this maximum and a reduction in intensity. Using the change in the surface plasmon band intensity at different Con A concentrations Fig. S3 was obtained, and the data for 3, 8, and 24 hours fitted with Hill functions. This yielded the dissociation rate constants displayed in Table S1. After 24 hours, the nanoparticle solution with the highest Con A concentration was subjected to an excess of D-mannose, and then sonicated, briefly. This resulted in the nanoparticles no longer agglomerating, and a regeneration of the surface plasmon resonance band in the UV-Vis spectra (Figure S4).

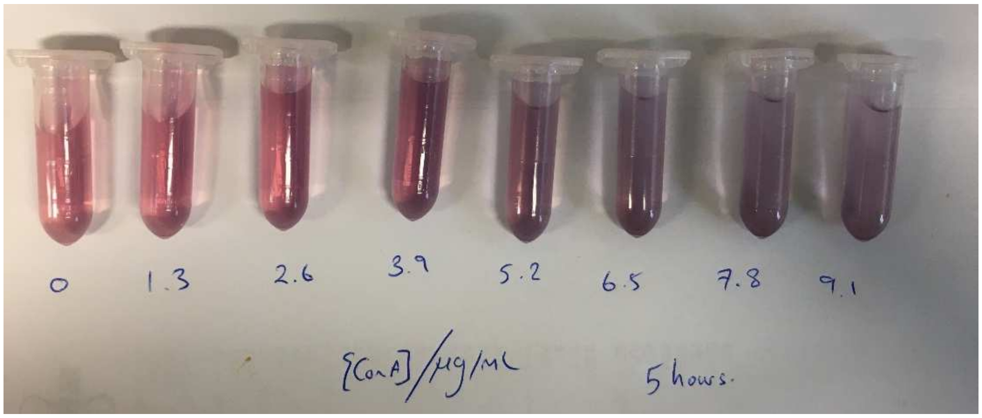

Figure S2: Photograph of mannose spin labelled Au nanoparticles with varying concentrations of Con A after 5 hours at room temperature.

Time after addition of Con A (h)	3	8	24
K_d (nM)	42.3	49.9	43.2

Table S1: Rate constants obtained from the Hill functions obtained from the data in Figure S3.

The changes in the UV-visible spectra upon titration with Con A could be fitted, giving an average apparent IC_{50} ≈ 45 nM (Figure S3) that is comparable to literature values.[9]

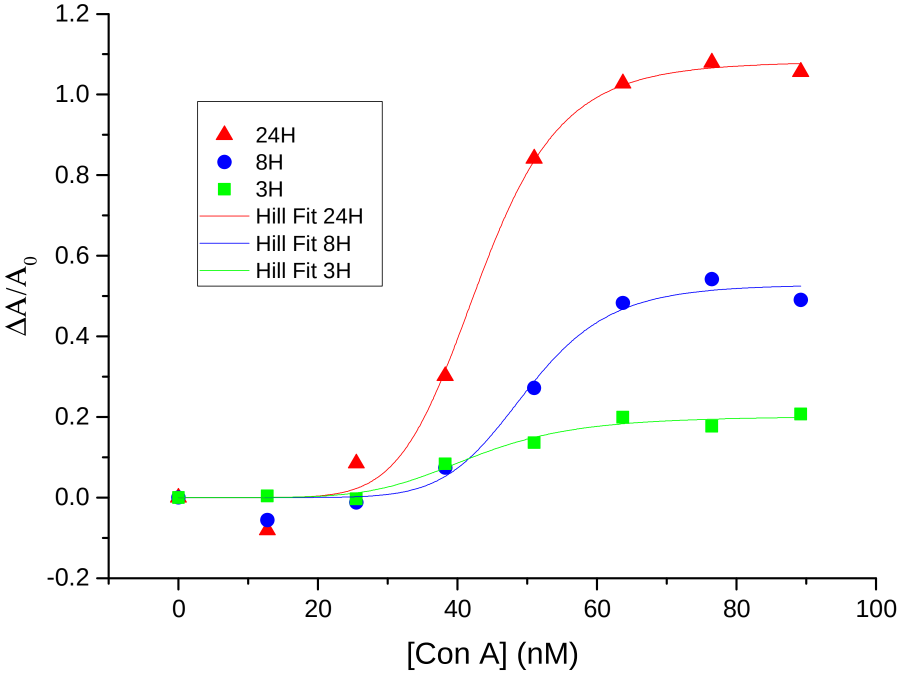

Figure S3: The change in the surface plasmon band (530 nm) intensity with Con A concentration at different times after lectin addition. A_0 is the surface plasmon resonance band intensity in the absence of Con A.

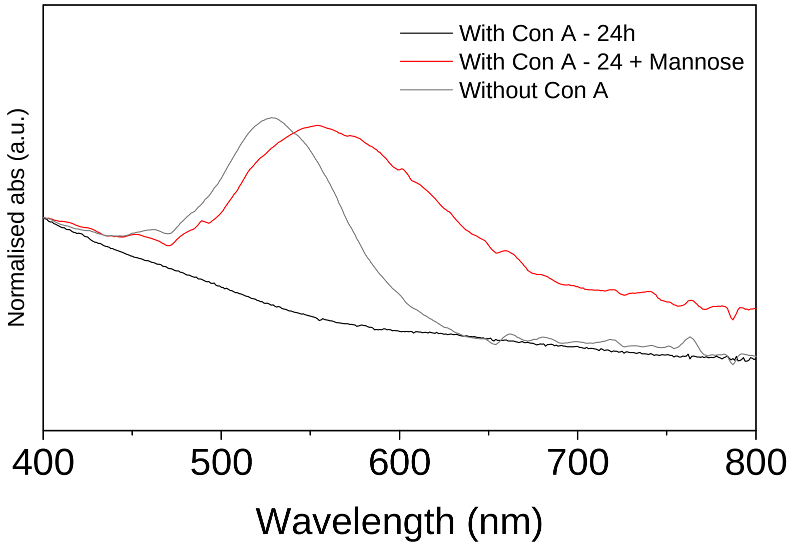

Figure S4: Normalised (at 400 nm) UV-Vis spectra of mannose spin labelled Au nanoparticles treated with Con A (9.1 µg/mL) for 24 hours (Black), treated with excess D-mannose (Red), and compared against Au nanoparticles with no Con A (Grey).

2. EPR and NMR

2.1 General EPR

CW X-band EPR spectra were acquired on a Bruker EMX micro instrument, equipped with a Bruker high Q X-band resonator. Bruker strong pitch (g = 2.0028) was used as a g-value reference. The nitroxide radicals and spin labelled nanoparticles were measured in 0.01 M potassium borate buffer (pH 9) at room temperature (295 K) using a flat cell (unless specified otherwise). 4-oxo-TEMPO was used as an intensity reference. Typical parameters for spin labelled gold nanoparticles were: modulation amplitude, 1.5 G; sweep width, 100 G; Scans, 250; attenuation, 20 mW.

2.2 Mannose/Con A EPR

2.2.1 Spin labelled mannose **4**

Con A exists as a homotetramer above pH 6, and each subunit contains both a Mn^{2+} and Ca^{2+} ion. However, both ions are not directly involved with glycoside binding in the active site, but are structurally essential for lectin to function. Despite the paramagnetic nature of Mn^{2+}, no observable EPR signal was observed at room temperature solution, which is consistent with previous literature, and is attributed to the flexible nature of the lectin Mn^{2+} site and possibly a fast relaxation time in solution.[10] The crystal structure of the methyl-mannose glycoside bound Con A complex is known in the literature;[11] using this crystal structure it was observed that the Mn^{2+} ion is >15 Å away from the mannose binding site, and even further from the predicted nitroxide location; attached *via* a linker, increasing the distance to over 20 Å. Therefore, it is unlikely that there will be significant broadening observed from dipolar coupling.

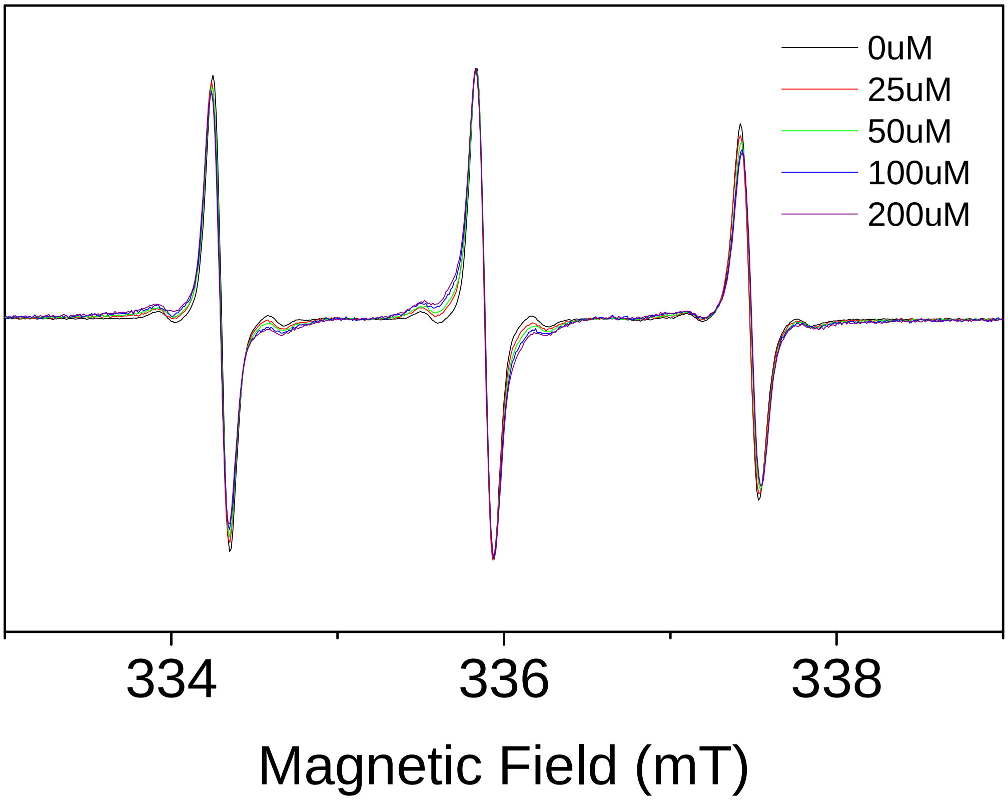

Figure S5: Normalized CW EPR spectra for spin labelled mannose aldehyde **4** (100 µM) with varying concentrations of Con A.

For the titration of the free spin labelled mannose **4** with Con A; a known quantity of a stock solution of **4** was added to 1 mL solutions of varying concentrations of Con A in 0.01 M potassium borate buffer (0-200 µM, created by adding aliquots of a 10 mg/mL stock solution of Con A), to create a 100 µM nitroxide concentration. These solutions were thoroughly vortexed, and left to reach equilibrium (5 mins). These samples were placed into capillary tubes and run using a Bruker EMX Micro spectrometer with an ER 4102ST X-band resonator.

The CW EPR spectra obtained were normalised to the center field line and compared (Figure S5). With increasing concentration of Con A an increased broadening of the resonances was observed, especially of the high field peak, along with a small increase in hyperfine splitting. This broadening has been attributed to the spin label entering a reduced motion environment on binding.

Simulations were created using EasySpin, a MATLAB toolbox, created by Stefan Stoll and Arthur Schweiger, allowing the simulation and fitting of a wide range of EPR spectra.[12] Simulations were created

using the data shown in Figure S5 for the mannose spin label **4** with and without Con A (Figure S6). A mixture of two species could be fitted to this data; a fast and a slow-motion species, corresponding to the free spin label, and bound label-Con A complex, respectively.

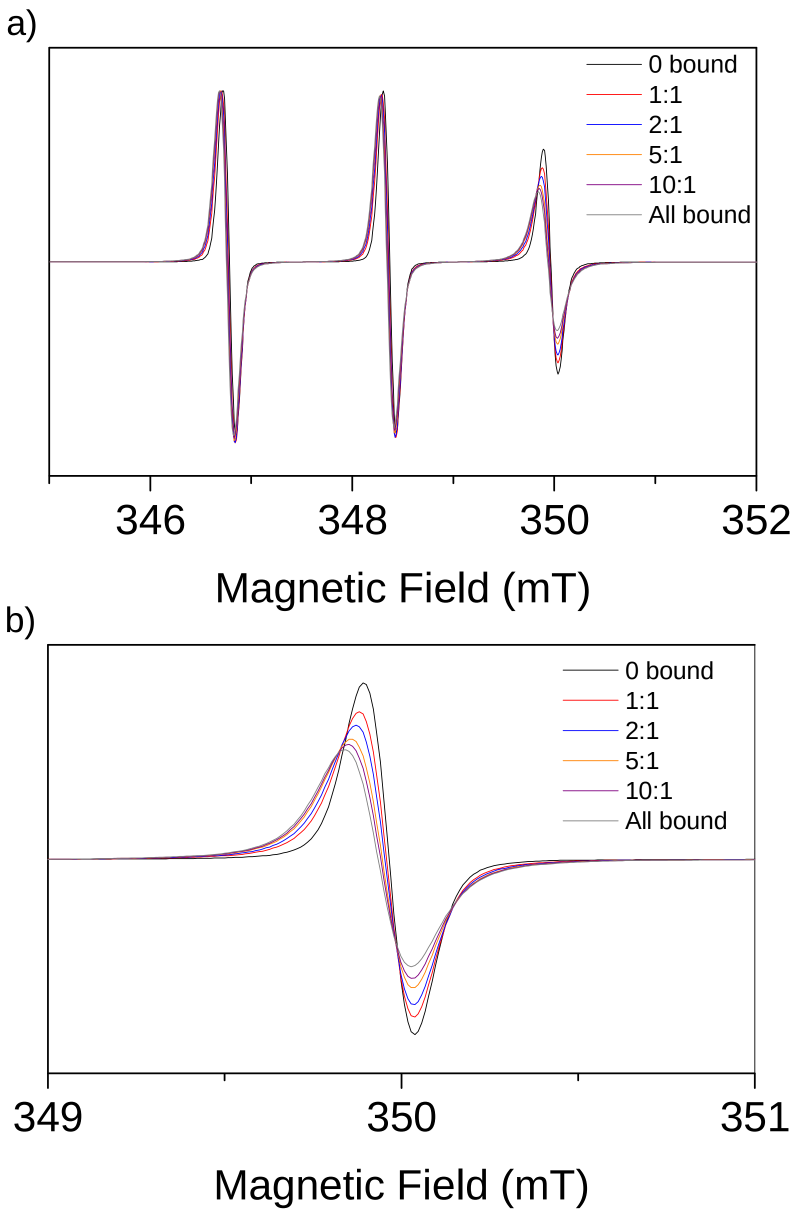

Figure S6: A simulation of a two component system, where there is relatively little broadening on binding a) full spectrum b) high field peak. The spectra are normalised to have the same mid field resonance height.

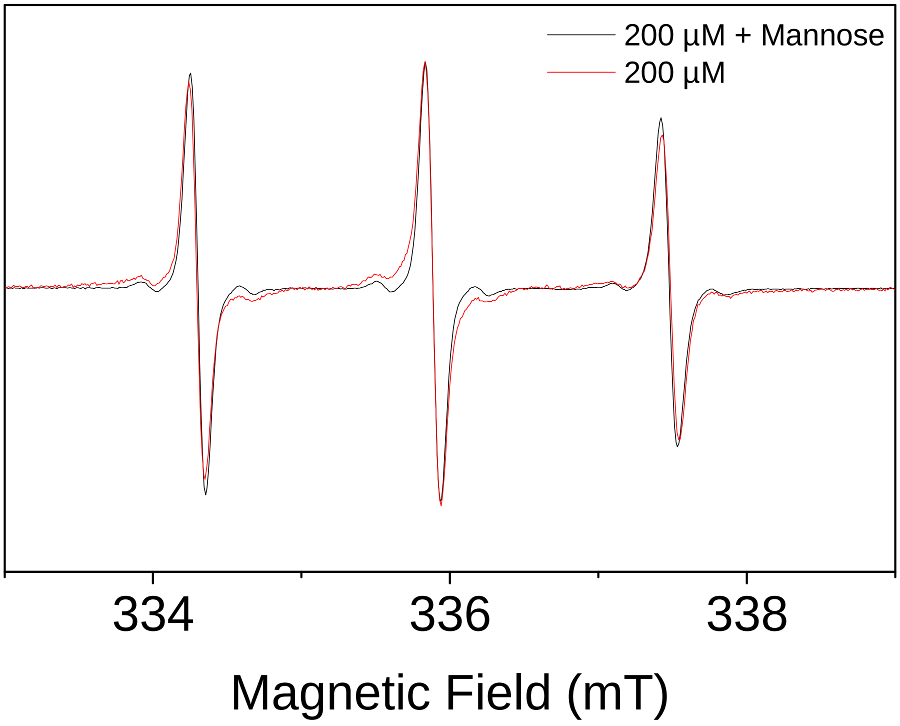

Figure S7: Normalized CW EPR spectra for spin labelled mannose aldehyde **4** (100 µM) with Con A (200 µM) before and after addition of mannose.

Subsequent addition of mannose to the mixture with the highest Con A concentration (200 µM) reduced both the broadening and hyperfine changes. This was ascribed to displacement of the spin labelled mannose by free mannose (Figure S7) and indicated that the observed changes in lineshape were due to Con A binding rather than changes in viscosity or polarity. On excess mannose addition, the broadening due to the binding of the spin label to Con A was significantly reduced, and a recovery of the centre resonance height was observed. This, again, indicated that the broadening is unlikely to be caused by changes in viscosity on the addition of Con A, as adding mannose to the solution is likely to increase the viscosity further, yet the broadening is reduced.

2.2.2 Mannose spin labelled nanoparticles

Con A was purchased from Sigma Aldrich as a lyophilised powder, and dissolved in a 0.01 M potassium borate buffer to make a stock solution. Mannose functionalised spin labelled nanoparticles (1

mL) were placed into 2 mL Eppendorf tubes, and varying amounts of a stock solution of Con A added, creating Con A concentrations between 10-200 µM. The nanoparticle solutions were vigorously stirred and left to equilibrate at room temperature for 10 minutes, before placing into an aqueous flat cell for EPR analysis.

When the normalised free spin label **4** CW EPR spectra was compared to the spin labelled nanoparticles (Figure S8) there was significant broadening of all three nitroxide resonances, indicating a reduced molecular motion of the spin label when on the surface of a nanoparticle. Addition of Con A (100 µM) to the mannose functionalised nanoparticles led to a visible colour change, and agglomeration of the nanoparticles. A change in the EPR line height ratios between the high and centre field resonances was also observed, similar to that seen for the free spin label, but was also seen for the low and centre field resonances, indicating that the rotational motion of the spin labelled mannose was perturbed by Con A, demonstrating binding.

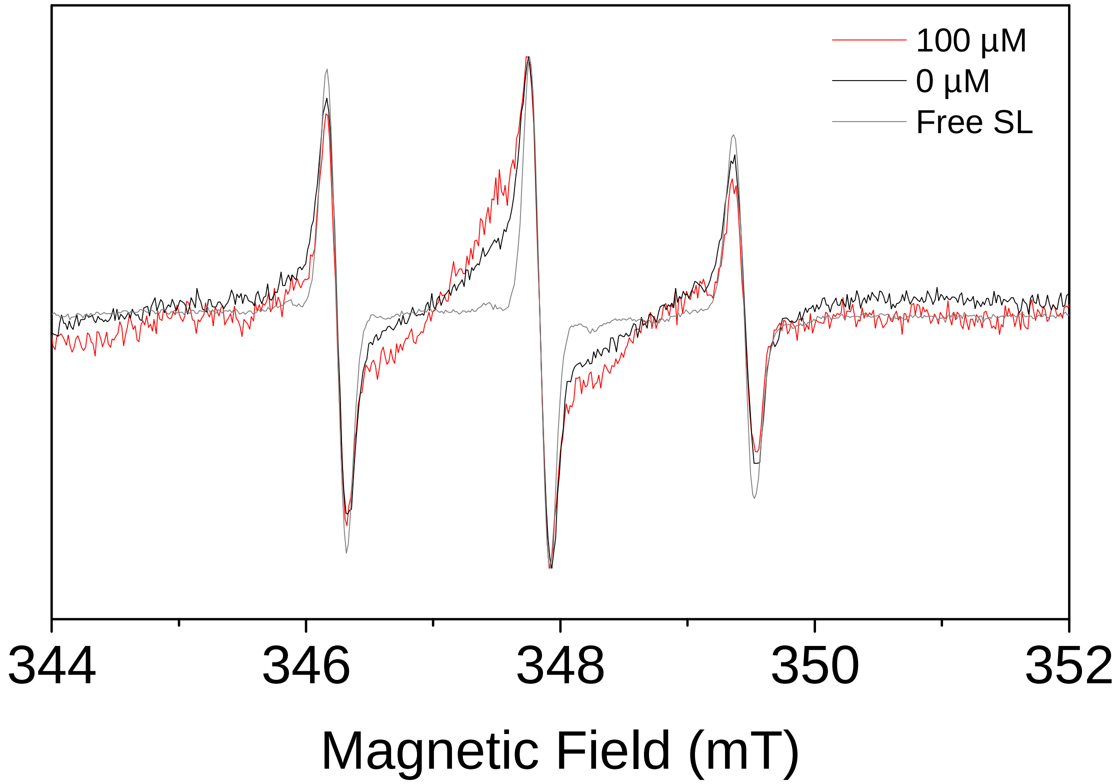

Figure S8: Normalised CW EPR spectra for spin labelled Au nanoparticles (1:1 functionalised alkyl thiols/PEG terminated alkyl thiols) with Con A (100 µM) in 0.01 M potassium borate buffer compared to the free spin label 5 in 0.01 M potassium borate buffer.

Agglomeration and subsequent precipitation led to a decay of the nitroxide spin label over time. This precipitation process had little effect on the shape of the EPR spectrum, as only a very small proportion of the nitroxide spin labels on the surface of the nanoparticles are bound by Con A, and most of the remaining spin labels are free to rotate/flex on the surface of the nanoparticle. EPR spectra were obtained for mannose functionalised nanoparticles with Con A (0-100 μM) which showed that there was a relationship between Con A concentration and the centre/high field ratio (Figure S9), although it was not possible to fit the data to a binding model.

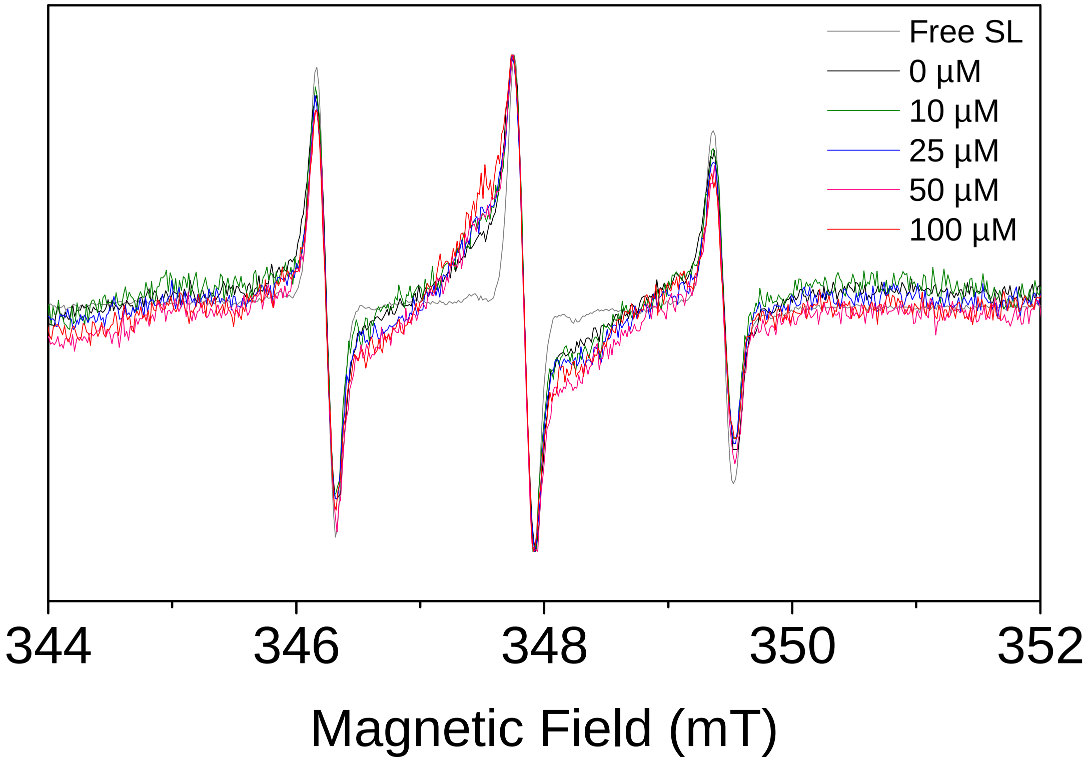

Figure S9: Normalised CW EPR spectra for spin labelled Au nanoparticles (1:1 functionalised alkyl thiols/PEG terminated alkyl thiols) with varying concentrations of Con A in 0.01 M potassium borate buffer compared to the free spin label **4** in 0.01 M potassium borate buffer.

CW EPR were also obtained for mannose spin labelled nanoparticles passivated with only 10 % carboxylic acid terminated thiols, treated with the same peptide coupling procedure as the 1:1 nanoparticles above (Figure S10). The spectra obtained showed less broadening than the 1:1

nanoparticles, with much better signal to noise, but also a smaller difference between the free spin label and the spin labelled nanoparticle. This reduction in broadening could be due to the close proximity of the spin labels to each other (in the 1:1 nanoparticle) causing exchange coupling, however, this would lead to the observation of a half-field transition at low temperature, which was not detected. Alternatively, the broadening could be attributed to the reduced molecular motion of the spin label in a more crowded environment. For a 1:1 nanoparticle, each spin label would have at least three nearest neighbours that are spin labelled alkyl thiols, while with the 10 % nanoparticle, this drops to less than one.

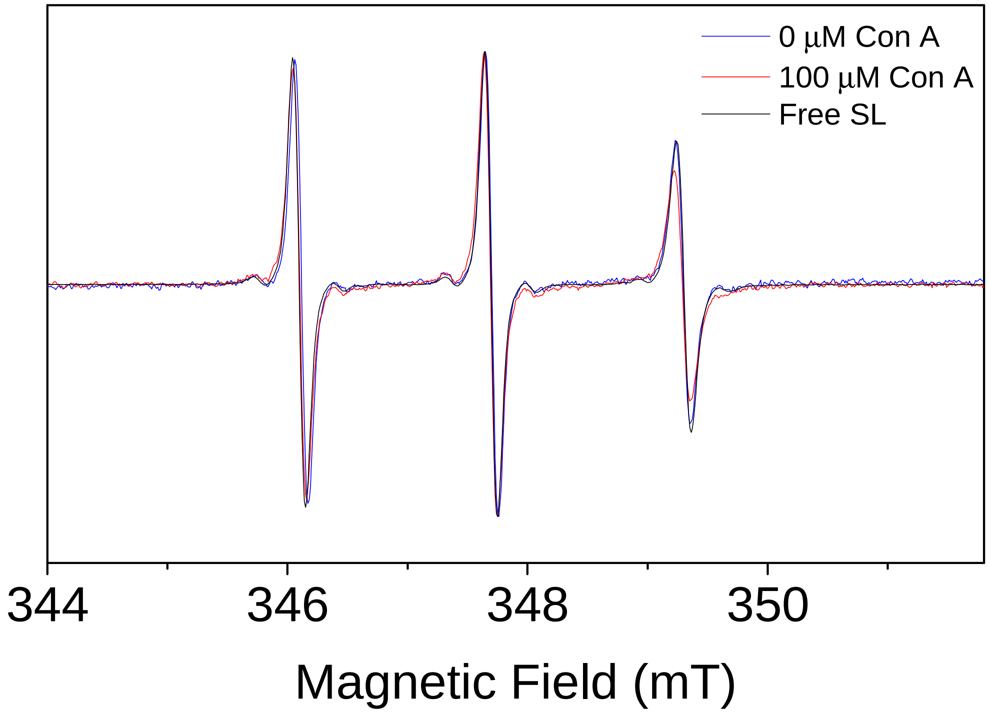

Figure S10: EPR spectra for spin labelled mannose functionalised nanoparticles. Normalised CW EPR spectra for spin labelled Au nanoparticles (1:9 functionalised alkylthiols/PEG terminated alkyl thiols) with and without Con A (100 µM).

Addition of Con A (100 µM) to the mannose functionalised nanoparticles once again led to a visible colour change, agglomeration of the nanoparticles, and a change in the ratio between the high and centre field line height, which appeared to be more pronounced than that seen for the 1:1 nanoparticles. Therefore, it appeared that the surface coverage of these nanoparticles was particularly important to control not just their bio-chemical properties, but also their physical/spectroscopic properties too.

The 10 % spin labelled nanoparticles could be simulated by one component, with a correlation time of $\log_{10}$ -9.3 (Figure S11). On addition of Con A, a mix of two components appeared; the sharp component was still there along with an axially broadened component ($\log_{Diff}$ = [1 2]*8), which led to the reduced line height of the low field resonance.

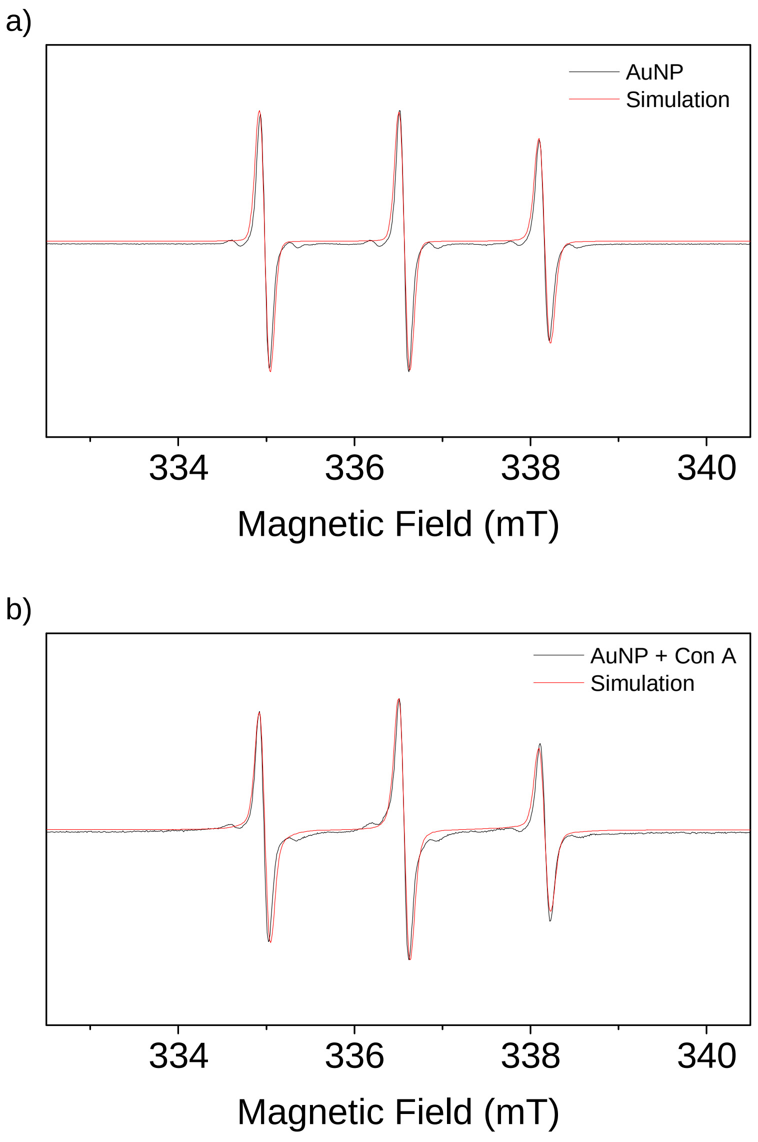

Figure S11: Simulations of the mannose functionalized nanoparticles **a)** without Con A and **b)** with Con A, compared with the obtained EPR spectra. The spectra are normalised to have the same mid field resonance height.

2.3 Biotin/Streptavidin EPR

2.3.1 Spin labelled biotin **5**

The biotin spin label **5** (2 µM) was treated with streptavidin (1.8 µM), and the CW EPR spectra obtained both with and without streptavidin. Using this data, simulations were created for the biotin spin label **5** with and without streptavidin. The free spin label could be simulated, with one component, with a rotational correlation time of 10^-9.98 s (Figure S12a). The biotin spin label with streptavidin, could be simulated with a combination of the free spin labelled simulation and a rather broad component, with a correlation time of 10^-8.3 s (Figure S12b).

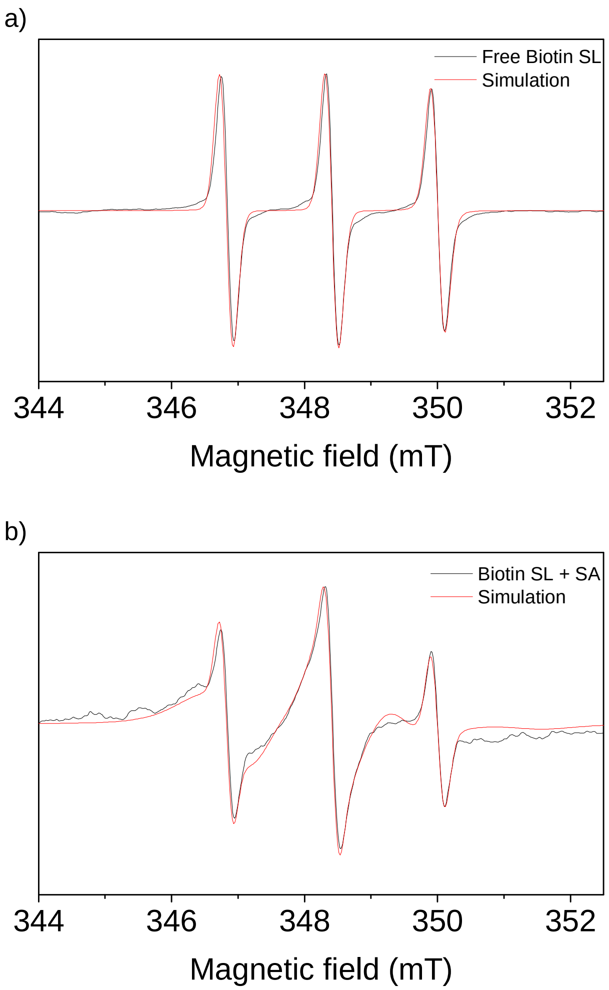

Figure S12: Simulations of the biotin spin label **5** without **a)** Streptavidin and **b)** with streptavidin (1.8 µM), compared with the obtained EPR spectra (biotin SL concentration 2 µM). The spectra are normalised to have the same mid field resonance height.

2.3.2 Biotin spin labelled nanoparticles

Unfortunately, the average molecular weight was unknown for the commercially available avidin-peroxidase, however, the molecular weights of both avidin and peroxidase are known, as well as the extinction coefficient for horseradish peroxidase. For the EPR titration studies, a 2 mg/mL solution of avidin-peroxidase was created, and small amounts added to 1 mL solutions of the biotinylated gold nanoparticles. In order to calculate the concentration of avidin-peroxidase binding sites, this 2 mg/mL solution was diluted to 0.2 mg/mL, and the UV-Vis spectrum obtained (Figure S13).

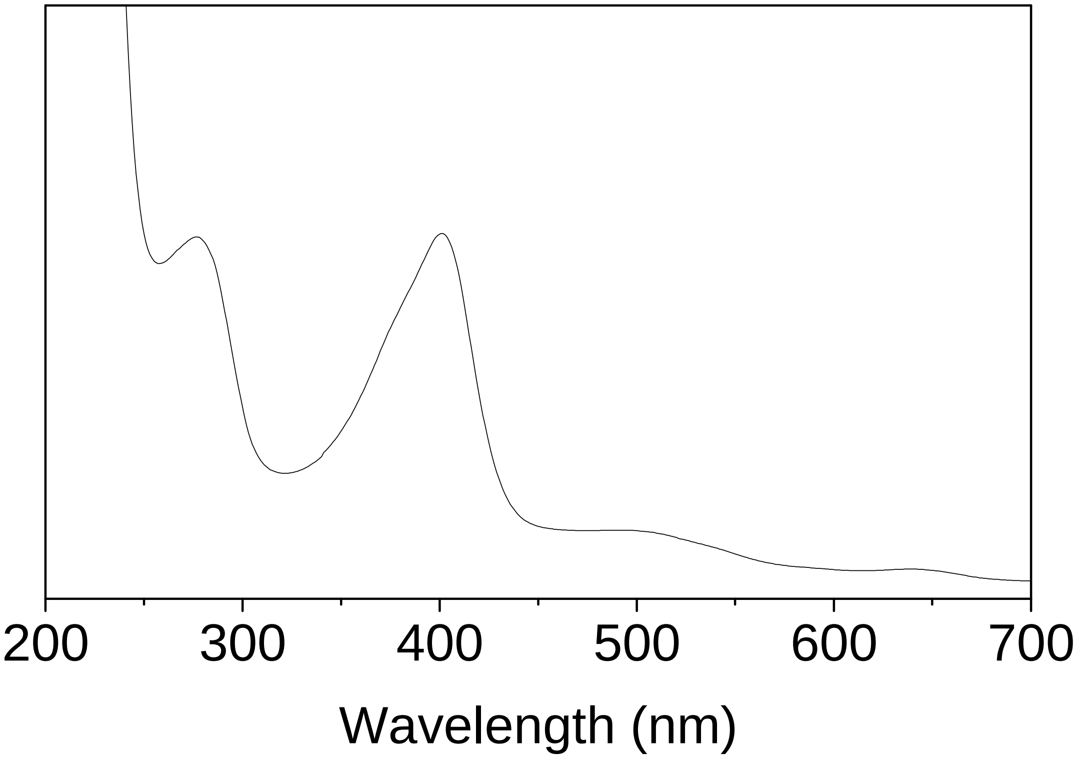

Figure S13: UV-Vis spectra of the avidin-peroxidase complex (0.2 mg/mL).

Using the absorbance at 403 nm (0.30647) and the extinction coefficient for horseradish peroxidase at 403 nm (1.02×10^5 $M^{-1}cm^{-1}$)[13] it was possible to obtain the HRP concentration (3.13×10^{-6} M).[14] Using the molecular weight of HRP (44,000 g/mol, obtained from the Sigma-Aldrich website) the concentration of HPR in mg/mL was 0.138 mg/mL. Therefore, assuming the rest of the mass is avidin, the concentration of avidin was 0.062 mg/mL (0.2 – 0.138). Given the molecular weight of the avidin tetramer is 68,000 (from Sigma-Aldrich website), the concentration of avidin in a 0.2 mg/mL solution of the avidin peroxidase

solution provided from Sigma-Aldrich, was 9.1×10^{-7} M. Therefore, the avidin binding site concentration of the 2 mg/mL avidin-peroxidase complex solution used in the titrations, was 3.64×10^{-5} M. Using this concentration, the concentration of the avidin-peroxidase/nanoparticle solutions were calculated, and are shown below in Table S2.

This UV-Vis data indicated that the Conjugate is probably a mixture with an average composition (avidin)$(HRP)_{3.44}$. This is higher than stated on the Sigma-Aldrich website: "extent of labelling: 0.7-2.0 mol peroxidase per mol avidin"

http://www.sigmaaldrich.com/catalog/product/sigma/a3151?lang=en&region=GB

However, the product specification sheet states: "Peroxidase Content 1.0 - 3.5 moles Peroxidase/mole Avidin."

added µL	Avidin-peroxidase binding site concentration (nM)
0.5	18.2
1	36.4
2	72.8
3	109.1
4	145.3
5	181.4
6	217.5
7	253.5
8	289.4
9	325.3
10	361.1

Table S2: Avidin-peroxidase binding site concentrations.

On addition of avidin-peroxidase, there was no observable change in the colour of the nanoparticle solution, and no observable agglomeration, even after 24+ hours with excess avidin peroxidase. Initially, this was prescribed to the addition of peroxidase to the avidin protein (as discussed above), however, on closer inspection of the literature,[15] it was revealed that agglomeration with avidin is slow and will only create small clusters, having no perceivable impact on the colour of the nanoparticle solution and no precipitation.

On binding, there was a significant broadening of the EPR spectrum; so significant, that it was almost impossible to detect with the particularly weak signal/noise that was obtained on measurement of the nanoparticles (Figure S13). With 1000 scans, it was possible to observe the remarkably broad component of the avidin-peroxidase bound nanoparticles, which showed that the observed EPR resonances barely overlap with the unbound nanoparticle resonances. This leds to the decreasing intensity of all three resonances of the spectra, which can be quantified to yield binding profiles (Figure S14).

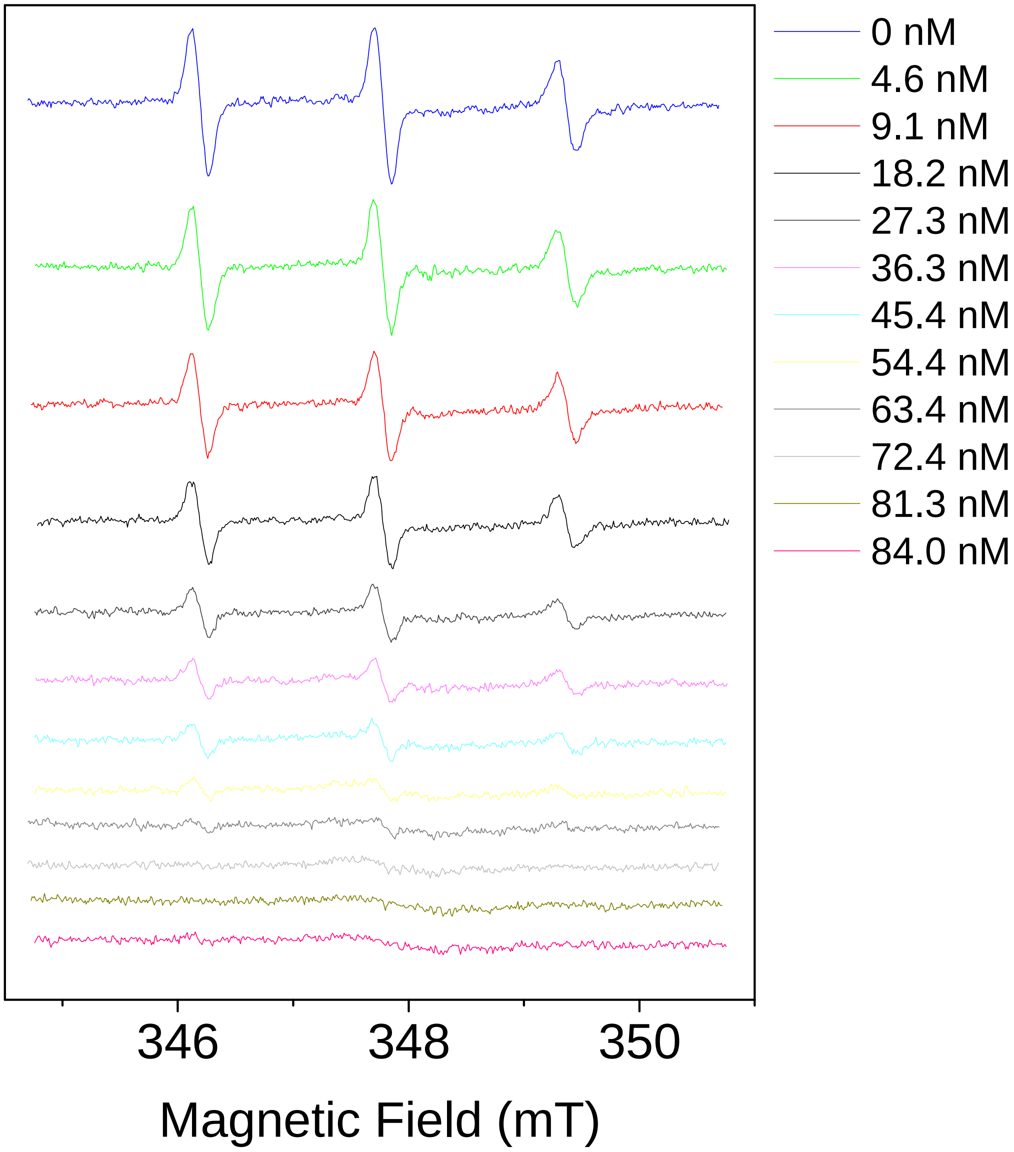

Figure S14: CW EPR spectra of biotin functionalized nanoparticles treated varying amounts of avidin peroxidase.

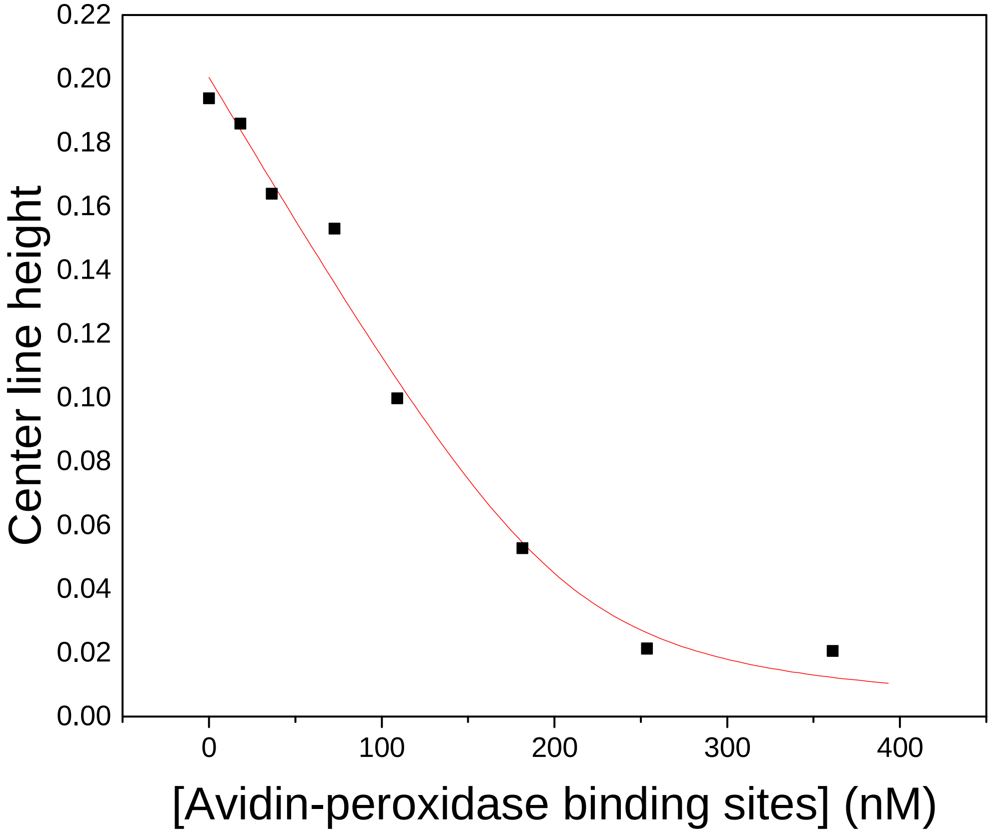

Figure S15: 1:1 Binding curve fitted to the change in line height of the mid field resonance of the biotin spin labelled nanoparticles against avidin-peroxidase concentration – repeated.

Using the avidin binding site concentrations shown in Table S2, and the centre line heights obtained from two separate experiments (Figure 4 in the main text corresponds to the data obtained in Figure S14), binding profiles were created. These profiles can be fitted by a 1:1 binding model using Dynafit, (Figure 4 and S15) to yield binding constants of 7.6 and 10.7 nM, respectively, with a spin label concentration ([L]) of 210 nM (which was calculated from the double integral of the EPR spectrum of the spin labelled gold nanoparticles). This indicated that the average binding constant for the biotinylated nanoparticles was somewhere in the region of 9.2 nM with a standard deviation of ± 1.55 nM.

2.3.3 Simulations of biotin spin labelled nanoparticles with streptavidin peroxidase

The biotin functionalised spin labelled nanoparticles could be simulated using the data shown in Figure 3. The spin labelled nanoparticles could be fitted by one component with an axial rotational correlation time of logDiff = [1 2] * 8.85 s (Figure S16a). The nanoparticles with avidin-peroxidase could roughly be fitted with a particularly broad component with a rotational correlation time of 10^-8.0 s, indicating that the label was in a hindered motion environment (Figure S16b). However, due to the significant broadening, it was difficult to obtain an exact fit, as the smaller peaks are lost in the noise. In contrast to the Con A binding, there was a significant broadening of the peaks, most likely due to the tight binding of the avidin-peroxidase leading to significantly reduced molecular motion.

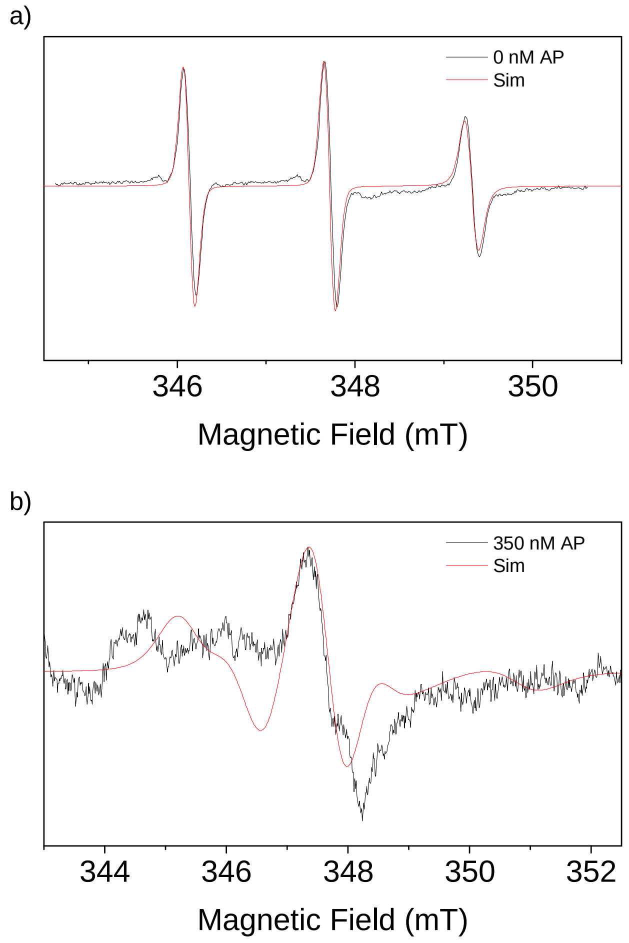

Figure S16: Simulations of the biotin spin labelled nanoparticles **a)** without and **b)** with avidin-peroxidase (350 nM), compared with the obtained EPR spectra. The spectra are normalised to have the same mid field resonance height

Using these two components, it is possible to create a simulated titration, to demonstrate how the line shape would be influenced by an increasing population of the protein-label complex (Figure S17). When the simulations were not normalised, we observed that the line heights were significantly smaller than those of the free spin label. It is likely that it would be difficult to detect the changes in the line height for the bound component experimentally (especially for the relatively low spin concentrations of nanoparticles). Interestingly, it appeared that there was little overlap for all three of the sharp component resonances by the broad component, indicating that these resonance line heights were dominated by the free spin label component, and therefore the equilibrium free spin label concentration. This meant that by quantifying the central line height while titrating avidin-peroxidase, it was possible to obtain a binding profile.

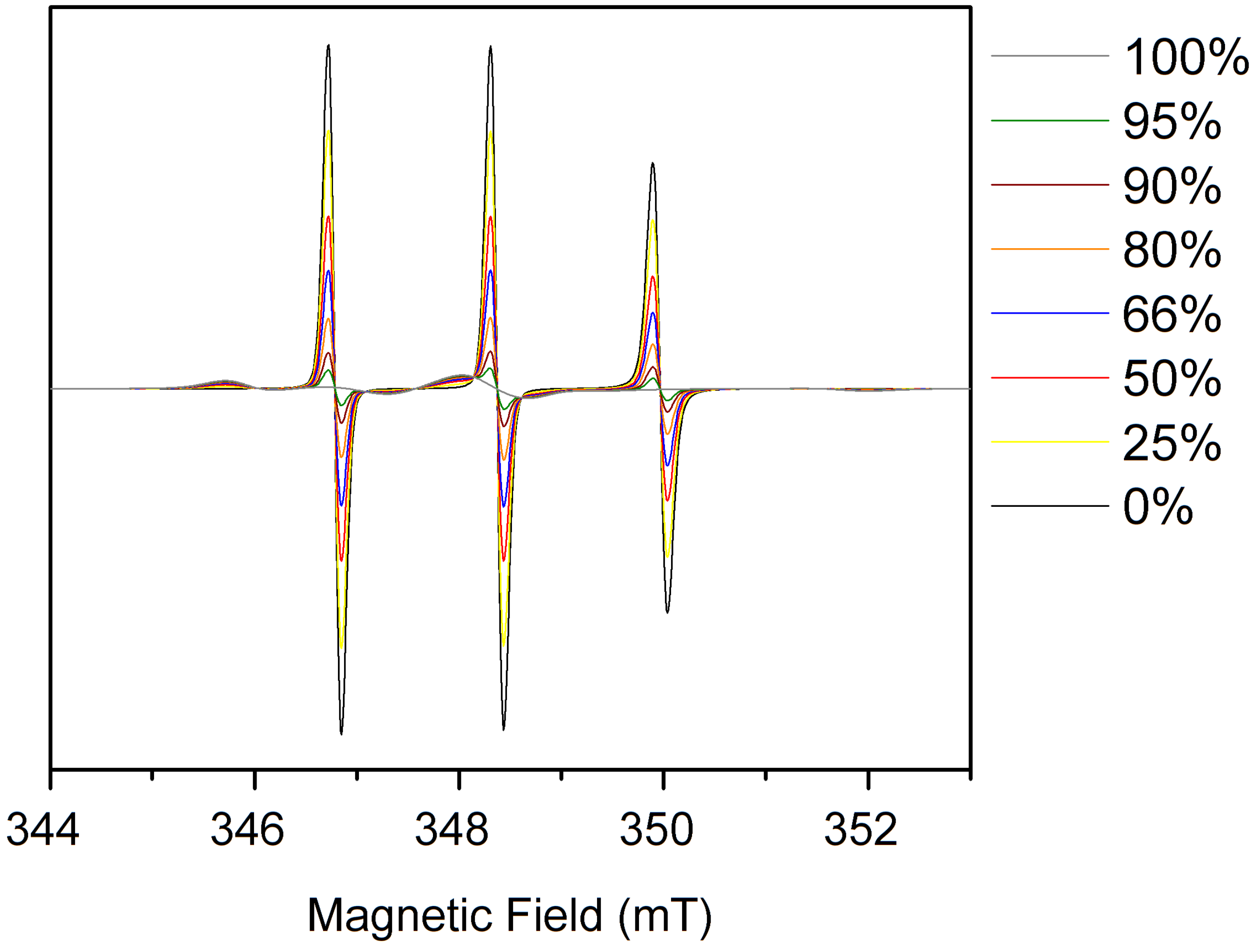

Figure S17: Simulations of a two component system (0-100 % bound), where there is significant broadening on binding.

2.3.4 Viscosity control

To further rule out viscosity as a source of the binding profile observed in Figure S7, a viscosity study was conducted with 100 µM spin labelled mannose **4** with varying amounts of glycerol, the viscosity of the solution increases, there is a reduction in tumbling rates of the nitroxide spin label, and a broadening of the resonances. As before, the ratio between the high field and central field resonance heights may be plotted against the % glycerol to give a binding profile (Figure S18).

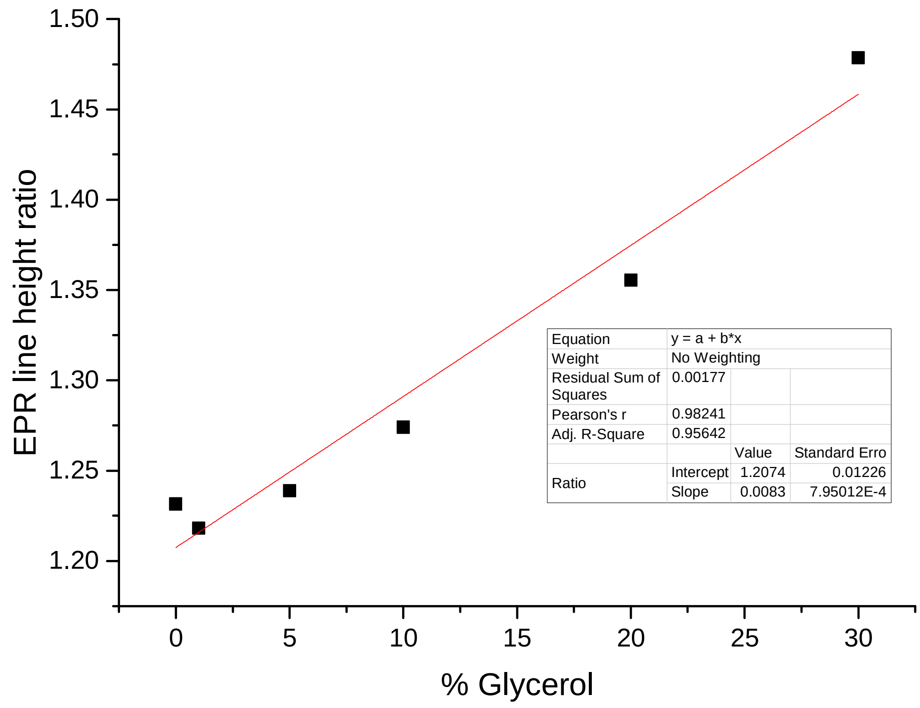

Figure S18: The change in relative line height between the high field and mid field resonances against % glycerol for 100 µM solutions of the nitroxide radical **4** in 0.01 M potassium borate buffer with various amounts of glycerol.

2.4 NMR of radicals

2.4.1 3-(2-Thioethyl-α-D-mannose)-4-formyl-2,2,5,5-tetramethyl-1H-pyrrol-1-yloxyl radical

1H (400 MHz, CD_3OD)

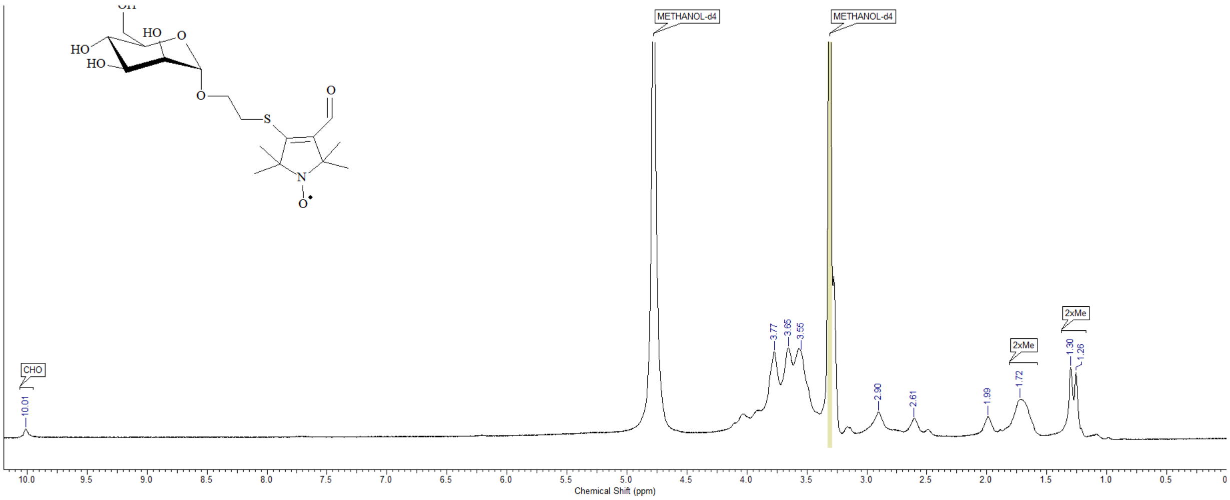

Note: Significant broadening was observed for the nitroxide radical **4**, due to the paramagnetic nature of nitroxide radicals. However, the broadened peaks for the aldehyde proton (δ 10.0) and the 4 methyl groups (δ 1.27 & 1.72) were visible.

3. Simulation Code

3.1.1 Mannose Spin Label with Con A (Figure S6)

```
Sys1.g = [2.0085, 2.006, 2.0021]; % Slow tumbling species
Sys1.lwpp = 0.3;
Sys1.Nucs = '14N';
Sys1.A = [20 20 94];
Sys1.logtcorr = -9.6;

Sys2.g = [2.008, 2.006, 2.0021]; % fast tumbling species
Sys2.lwpp = 0.12;
Sys2.Nucs = '14N';
Sys2.A = [20 20 94];
Sys2.logtcorr = -9.8;
```

```
Exp.mwFreq = 9.778248;
Exp.CenterSweep = [348 10]; % in mT
[B,spec1] = chili(Sys1,Exp); % Slow tumbling species
[B,spec2] = chili(Sys2,Exp); % fast tumbling species

[sumspec] = x*[spec1] + x*[spec2] % Set bound ratio
plot(B,sumspec);
```

3.1.2 Biotin Spin Label with Streptavidin (Figure S12)

```
Sys1.g = [2.0085, 2.006, 2.0021]; % fast tumbling species
Sys1.lwpp = 0.2;
Sys1.Nucs = '14N';
Sys1.A = [20 20 94];
Sys1.logtcorr = -9.98;


Sys2.g = [2.008, 2.006, 2.0021]; % Slow tumbling species
Sys2.lwpp = 0.2;
Sys2.Nucs = '14N';
Sys2.A = [20 20 94];
Sys2.logtcorr = -8.3;

Exp.mwFreq = 9.780195;
Exp.CenterSweep = [348 20]; % in mT
Exp.Mod = 2

[B1,spec1] = chili(Sys1,Exp);
[B2,spec2] = chili(Sys2,Exp);

[sumspec] = 0.10*[spec1]+ 1*[spec2] % Set bound ratio

[sumspec] = ([sumspec]/max(sumspec)); % Normalise

plot(B2,sumspec);
```

3.1.3 Mannose Spin Labelled Nanoparticles (Figure S11)

```
Sys1.g = [2.0085, 2.006, 2.0021]; % Sharp component
Sys1.lwpp = 0.12;
Sys1.Nucs = '14N';
Sys1.A = [20 20 94];
Sys1.logDiff = 9.3

Sys2.g = [2.008, 2.006, 2.0021]; % broader component – axial rotational diff
Sys2.lwpp = 0.12;
Sys2.Nucs = '14N';
Sys2.A = [20 20 94];
Sys2.logDiff = [1 2]*8

Exp.mwFreq = 9.447744;
Exp.CenterSweep = [336 10]; % in mT

[B1,spec1] = chili(Sys1,Exp);
```

```
[B2,spec2] = chili(Sys2,Exp);

[simspec] = x*[spec1]+x*[spec2] % ratio of sharp vs broad component

plot(B1, simspec);
```

3.1.4 Biotin Spin Labelled Nanoparticles (Figure S16 and S17)

```
Sys1.g = [2.0085, 2.006, 2.0021]; % Slow tumbling species
Sys1.lwpp = 0.3;
Sys1.Nucs = '14N';
Sys1.A = [20 20 94];
Sys1.logtcorr = -8.0;

Sys2.g = [2.0085, 2.006, 2.0021]; % fast tumbling species
Sys2.lwpp = 0.12;
Sys2.Nucs = '14N';
Sys2.A = [20 20 94];
Sys2.logDiff = [1 2]*8.85;

Exp.mwFreq = 9.778248;

Exp.CenterSweep = [348 10]; % in mT
[B,spec1] = chili(Sys1,Exp); % Slow tumbling species
[B,spec2] = chili(Sys2,Exp); % fast tumbling species

[sumspec] = x*[spec1] + x*[spec2] % Set bound ratio

[sumspec] = ([sumspec]/max(sumspec)); % Normalise

plot(B,sumspec);
```

4. References

(1) Chudinov, A. V.; Rozantsev, É. G.; Rozynov, B. V. *Bull. Acad. Sci. USSR Div. Chem. Sci.* **1983**, *32* (2), 356–360.

(2) Chudinov, A. V.; Rozantsev, É. G.; Rozynov, B. V. *Bull. Acad. Sci. USSR Div. Chem. Sci.* **1983**, *32* (2), 370–375.

(3) García-Barrientos, A.; García-López, J. J.; Isac-García, J.; Ortega-Caballero, F.; Uriel, C.; Vargas-Berenguel, A.; Santoyo-González, F. *Synthesis* **2001**, *2001* (07), 1057–1064.

(4) Wang, T.-P.; Chiou, Y.-J.; Chen, Y.; Wang, E.-C.; Hwang, L.-C.; Chen, B.-H.; Chen, Y.-H.; Ko, C.-H. *Bioconjug. Chem.* **2010**, *21* (9), 1642–1655.

(5) Wang, T.-P.; Ko, N. C.; Su, Y.-C.; Wang, E.-C.; Severance, S.; Hwang, C.-C.; Shih, Y. T.; Wu, M. H.; Chen, Y.-H. *Bioconjug. Chem.* **2012**, *23* (12), 2417–2433.

(6) Techane, S. D.; Gamble, L. J.; Castner, D. G. *J. Phys. Chem. C* **2011**, *115* (19), 9432–9441.

(7) Haiss, W.; Thanh, N. T. K.; Aveyard, J.; Fernig, D. G. *Anal. Chem.* **2007**, *79* (11), 4215–4221.

(8) Otsuka, H.; Akiyama, Y.; Nagasaki, Y.; Kataoka, K. *J. Am. Chem. Soc.* **2001**, *123* (34), 8226–8230.

(9) Richards, S.-J.; Otten, L.; Gibson, M. I. *J Mater Chem B* **2016**, *4* (18), 3046–3053.

(10) George H. Reed; Cohn, M. *J. Biol. Chem.* **1970**, *245* (3), 662–667.

(11) Naismith, J. H.; Field, R. A. *J. Biol. Chem.* **1996**, *271* (2), 972–976.

(12) Stoll, S.; Schweiger, A. *J. Magn. Reson.* **2006**, *178* (1), 42–55.

(13) Gasyna, Z. *FEBS Lett.* **1979**, *106* (1), 213–218.

(14) Rota, C.; Chignell, C. F.; Mason, R. P. *Free Radic. Biol. Med.* **1999**, *27* (7–8), 873–881.

(15) Swift, J. L.; Heuff, R.; Cramb, D. T. *Biophys. J.* **2006**, *90* (4), 1396–1410.
